# CPT1A in AgRP neurons is required for sex-dependent regulation of feeding and thirst

**DOI:** 10.1186/s13293-023-00498-8

**Published:** 2023-03-25

**Authors:** Sebastián Zagmutt, Paula Mera, Ismael González-García, Kevin Ibeas, María del Mar Romero, Arnaud Obri, Beatriz Martin, Anna Esteve-Codina, M. Carmen Soler-Vázquez, Marianela Bastias-Pérez, Laia Cañes, Elisabeth Augé, Carme Pelegri, Jordi Vilaplana, Xavier Ariza, Jordi García, José Martinez-González, Núria Casals, Miguel López, Richard Palmiter, Elisenda Sanz, Albert Quintana, Laura Herrero, Dolors Serra

**Affiliations:** 1 Department of Biochemistry and Physiology, School of Pharmacy and Food Sciences, Universitat de Barcelona, Av. Joan XXIII, 27-31, 08028 Barcelona, Spain; 2 Institut de Biomedicina de la Universitat de Barcelona (IBUB), Universitat de Barcelona, Barcelona, Spain; 3 Centro de Investigación Biomédica en Red de Fisiopatología de la Obesidad y la Nutrición (CIBEROBN), Instituto de Salud Carlos III, Madrid, Spain; 4 NeurObesity Group, Department of Physiology, CIMUS, University of Santiago de Compostela-Instituto de Investigación Sanitaria, Santiago de Compostela, Spain; 5 Neuronal Control of Metabolism Laboratory, Institut d’Investigacions Biomèdiques August Pi i Sunyer (IDIBAPS), Barcelona, Spain; 6 CNAG-CRG, Centre for Genomic Regulation, Barcelona Institute of Science and Technology, 08028 Barcelona, Spain; 7 Universitat Pompeu Fabra (UPF), Barcelona, Spain; 8 Instituto de Investigaciones Biomédicas de Barcelona (IIBB-CSIC), Barcelona, Spain; 9 CIBER de Enfermedades Cardiovasculares (CIBERCV), Instituto de Salud Carlos III, Madrid, Spain; 10 Instituto de Investigación Biomédica Sant Pau (IIB-Sant Pau), Barcelona, Spain; 11 Biomedical Research Networking Centre in Neurodegenerative Diseases (CIBERNED), Madrid, Spain; 12 Institute of Neurosciences of the Universitat de Barcelona, Barcelona, Spain; 13 Department of Inorganic & Organic Chemistry, Faculty of Chemistry, Institut de Biomedicina de la Universitat de Barcelona (IBUB), Universitat de Barcelona, Barcelona, Spain; 14 Department of Basic Sciences, Faculty of Medicine & Health Sciences, Universitat Internacional de Catalunya, Sant Cugat del Vallès, Spain; 15 Department of Biochemistry, Howard Hughes Medical Institute, University of Washington, Seattle, WA USA; 16 Department of Cell Biology, Physiology and Immunology, Universitat Autònoma de Barcelona, Bellaterra, Spain; 17 Institut de Neurociències, Universitat Autònoma de Barcelona, Bellaterra, Spain

**Keywords:** CPT1A, Fatty acid metabolism, AgRP neurons, Energy balance, Food intake, Thirst

## Abstract

**Background:**

Fatty acid metabolism in the hypothalamus has an important role in food intake, but its specific role in AgRP neurons is poorly understood. Here, we examined whether carnitinea palmitoyltransferase 1A (CPT1A), a key enzyme in mitochondrial fatty acid oxidation, affects energy balance.

**Methods:**

To obtain *Cpt1a*KO mice and their control littermates, *Cpt1a*^(flox/flox)^ mice were crossed with tamoxifen-inducible AgRP^CreERT2^ mice. Food intake and body weight were analyzed weekly in both males and females. At 12 weeks of age, metabolic flexibility was determined by ghrelin-induced food intake and fasting–refeeding satiety tests. Energy expenditure was analyzed by calorimetric system and thermogenic activity of brown adipose tissue. To study fluid balance the analysis of urine and water intake volumes; osmolality of urine and plasma; as well as serum levels of angiotensin and components of RAAS (renin–angiotensin–aldosterone system) were measured. At the central level, changes in AgRP neurons were determined by: (1) analyzing specific AgRP gene expression in RiboTag–*Cpt1a*KO mice obtained by crossing *Cpt1a*KO mice with RiboTag mice; (2) measuring presynaptic terminal formation in the AgRP neurons with the injection of the AAV1*-EF1a-DIO-synaptophysin-GFP* in the arcuate nucleus of the hypothalamus; (3) analyzing AgRP neuronal viability and spine formations by the injection AAV9*-EF1a-DIO-mCherry* in the arcuate nucleus of the hypothalamus; (4) analyzing in situ the specific AgRP mitochondria in the ZsGreen-*Cpt1a*KO obtained by breeding ZsGreen mice with *Cpt1a*KO mice. Two-way ANOVA analyses were performed to determine the contributions of the effect of lack of CPT1A in AgRP neurons in the sex.

**Results:**

Changes in food intake were just seen in male *Cpt1a*KO mice while only female *Cpt1a*KO mice increased energy expenditure. The lack of *Cpt1a* in the AgRP neurons enhanced brown adipose tissue activity, mainly in females, and induced a substantial reduction in fat deposits and body weight. Strikingly, both male and female *Cpt1a*KO mice showed polydipsia and polyuria, with more reduced serum vasopressin levels in females and without osmolality alterations, indicating a direct involvement of *Cpt1a* in AgRP neurons in fluid balance. AgRP neurons from *Cpt1a*KO mice showed a sex-dependent gene expression pattern, reduced mitochondria and decreased presynaptic innervation to the paraventricular nucleus, without neuronal viability alterations.

**Conclusions:**

Our results highlight that fatty acid metabolism and CPT1A in AgRP neurons show marked sex differences and play a relevant role in the neuronal processes necessary for the maintenance of whole-body fluid and energy balance.

**Supplementary Information:**

The online version contains supplementary material available at 10.1186/s13293-023-00498-8.

## Highlights

- Fatty acid metabolism and CPT1A in AgRP neurons show marked sex-dependent differences in the control of feeding.
- *Cpt1a* gene deletion in AgRP neurons increases energy expenditure in females but not in males.
- CPT1A in AgRP neurons is involved in the control of thirst and fluid homeostasis.
- *Cpt1a* gene deletion in AgRP neurons induces morphological, mitochondrial, and gene expression alterations in a sex-dependent manner.

**Supplementary Information:**

The online version contains supplementary material available at 10.1186/s13293-023-00498-8.

## Background

Food intake and whole-body energy balance are regulated by the brain through a sophisticated neuronal network located mostly in the hypothalamus. In particular, the hypothalamic arcuate nucleus (ARC) is a fundamental sensor of the hormones and nutrients that indicate the energy status of the organism. The ARC contains two populations of neurons with opposite functions: anorexigenic proopiomelanocortin (POMC)-expressing neurons and orexigenic agouti-related protein (AgRP)-expressing neurons hypothalamus [1–4]. AgRP neurons promote a potent orexigenic response by opposing the actions of POMC neurons, in part through the release of the AgRP neuropeptide, a competitive inhibitor of melanocortin receptors [5, 6]. It also has an important effect on energy expenditure (EE) via modifying the activity of the sympathetic nervous system (SNS) [3]. The outflow onto peripheral tissues leads to coordination of overall body metabolism, including adipose tissues, liver and other tissues that respond to AgRP neuronal activity. For that reason, the action of AgRP neurons on energy balance should be studied beyond the acute control of food intake [7]. Although our understanding of the AgRP neuronal mechanism controlling feeding behavior has expanded immeasurably, few studies have explored sex-dependent regulatory mechanisms, especially considering the sex differences in calorie intake and body weight [8–10] and new studies are necessary to reveal sex differences in neuronal mechanisms that control energy balance.

In addition to food consumption, another physiological need essential for survival is thirst. Several brain areas involved in the control of fluid balance and thirst have been already elucidated [11–19], including the lamina terminalis, comprising the subfornical organ (SFO), organum vasculosum of the lamina terminalis (OVLT), and the median preoptic nucleus (MnPO). Neurons from these nuclei project to paraventricular nucleus of hypothalamus (PVH) where the neurons that produce vasopressin (VP, also known anti-diuretic hormone, ADH) are located [20]. The involvement of AgRP neurons in thirst behavior is poorly understood. It is known that AgRP activation induces water intake in the presence of food, but this activation is not sufficient to promote water intake independent of food consumption [21], suggesting that food and drink behavior are tightly coordinated.

Lipid metabolism has emerged as a key regulator of energy balance [22–24]. Although the oxidation of fatty acids (FAs) has been explored little in neurons, FAs undoubtedly have an important role through their structural role in biological membranes, their participation as a messenger in neuronal signaling pathways and their involvement in the energy supply [25, 26]. Here, we explore some key aspects of FA utilization in the control of energy balance and raise new questions on whether FAs could be also a signal for hypothalamic neurons. It is well documented that most enzymes in FA metabolic pathways are expressed in the hypothalamus, including AMP-activated protein kinase (AMPK), acetyl-CoA carboxylase (ACC) carnitine palmitoyltransferase 1 (CPT1), fatty acid synthase (FAS), and malonyl-CoA decarboxylase (MCD) [27, 28]. Of note, fasting stimulates hypothalamic AMPK activity and inhibits ACC and FAS activities, whereas re-feeding induces the opposite [26, 29–31]. Moreover, the pharmacological and genetic manipulation of some of these genes/proteins has a profound impact on food intake and whole-body energy homeostasis [32–34].

Here, we focused on CPT1A, a key regulatory enzyme localized in the outer mitochondrial membrane that is involved in the uptake of long-chain fatty acids (LCFAs) into the mitochondrial matrix for entry into the β-oxidation spiral [35]. Although this isoform is expressed mainly in the liver, there is evidence that its expression in the brain could play an important role in peripheral metabolism. Previous results from our laboratory showed that the long-term expression of a permanently activated CPT1A isoform in the hypothalamus triggers hyperphagia, leading to an overweight state [36]. Consistent with these results, the intracerebroventricular administration of 3-carboxy-4-alkyl-2-methylenebutyrolactone (C75), a synthetic inhibitor of fatty acid synthase (FAS) and CPT1A activity, has been reported to reduce body weight and food intake by altering the melanocortin system [37–39].

Most of the studies in this field lack cell type-specificity and it is still unclear whether lipid metabolism specifically regulates the physiological features modulated by AgRP neurons. To answer this question, we developed an adult transgenic mouse model lacking CPT1A in their AgRP neurons. Here, we report that AgRP neurons require CPT1A to regulate some aspects of energy balance, since female and male *Cpt1a*KO mice showed reduced body weight gain compared with their control littermates. Notably, this regulation varied depending on the sex. We also provide evidence that the metabolic changes induced by CPT1A deficiency in AgRP neurons modify the fluid balance. Our results suggest that CPT1A in AgRP neurons affect food intake and the peripheral energy balance in a sex-dependent manner, highlighting this enzyme as a possible target for therapeutic strategies that aim to decrease body weight and fight obesity.

## Materials and methods

### Mice

Mice were kept in the Unitat d’Experimentació Animal facilities of the Universitat de Barcelona under standard laboratory conditions with free access to standard chow diet (Harlan Ibérca, ref. 2014) and water at 22 ± 2 °C and 60% humidity in a 12-h light/dark cycle. Mice were housed in groups to prevent isolation stress unless otherwise stated. Mice animal welfare was regularly monitored. Experiments were performed with 8- to 25-week-old male and female mice.

To evaluate the role of FA metabolism in AgRP neurons, we generated the time-dependent conditional *Cpt1a*KO mouse model. *Cpt1a*^(flox/flox)^ mice [40] and tamoxifen-inducible AgRP^CreERT2^ mice [41] were bred to generate (*Cpt1a*^(flox/flox)^; *Agrp*^*CreERT2*^) mice and their control littermates (*Cpt1a*^(+/+)^; AgRP^CreERT2^), which were called *Cpt1a*KO and control mice, respectively. All animals were on the C57BL/6J background. To evaluate AgRP neuron-specific RNA expression, we took advantage of the RiboTag method [42]. We bred the RiboTag mouse line with *Cpt1a*KO mice to generate RiboTag;*Cpt1a*^(flox/flox)^; AgRP^CreERT2^ (RiboTag-*Cpt1a*KO) mice and their RiboTag;*Cpt1a*^(+/+)^; AgRP^Cre ER^^T2^ control littermates. These transgenic mice expressed, in an inducible manner, epitope-tagged ribosomal protein L22-hemagglutinin (RPL22-HA) in only the AgRP neurons.

To study mitochondrial morphology specifically in the AgRP neurons, we generated a Rosa26-flox-stop-MLS-ZsGreen mice (ZsGreen mice). A Cre-dependent mitochondrial-ZsGreen-expressing mice that allows targeting of ZsGreen to the mitochondrial matrix. The mitochondrial translocation sequence (MLS) from the Ndufs4 gene was fused in frame at the N-terminus of ZsGreen, which was then inserted 3′ of a floxed Pgk-neomycin resistance (Neo) gene (for positive selection) in a transfer plasmid. The MLS-ZsGreen-floxed Pgk-Neo region was excised and inserted into a targeting vector that has the CMV-chicken β-Actin promoter (CBAp) inserted at the transcription start site of the Gt(Rosa26)Sor gene with 7.7 kb of 5′ flanking and 4.1 kb of 3′ flanking Gt(Rosa26)Sor sequence and a Pgk-DTa gene for negative selection. This construct was linearized and electroporated into G4 embryonic stem (ES) cells. Correct gene targeting was determined by Southern blot of DNA digested with Nde1 using a probe that lies outside of the targeting vector; 24 of 84 clones analyzed were correctly targeted. ES cells from one correctly targeted clone were injected into blastocysts of C57BL/6 recipients and chimeric pups were bred with C57BL/6 mice. The action of Cre recombinase deletes Pgk-Neo and allows expression of ZsGreen that is targeted to the mitochondria. We bred ZsGreen mice with *Cpt1a*KO mice to generate ZsGreen; *Cpt1a*^(flox/flox)^; AgRP^Cre ERT2^ (ZsGreen-*Cpt1a*KO) mice and their ZsGreen-*Cpt1a*^(+/+)^; AgRP^CreERT2^ control littermates.

To induce Cre-ER^T2^ expression and avoid the possible toxic effect of tamoxifen, adult mice (8 weeks old) were intraperitoneally (i.p.) injected with five doses of tamoxifen (150 mg/kg of body weight; Sigma, MO, USA) solved in corn oil. The first two injections were combined with 24 h of food deprivation (separated by 48 h) to enhance *Agrp* promoter activity. The last three injections were administered daily with ad libitum access to food and water. Control mice were also injected with tamoxifen solved in corn oil. All studies used age-matched littermates, randomly assigned to experimental groups.

Mice were genotyped by polymerase chain reaction (PCR) using the primer listed in Additional file 7: Table S1. Genomic DNA was extracted from ear samples using the QuickExtract™ DNA Extraction solution, following the manufacturer’s instructions. Each polymerase chain reaction (PCR) was conducted in a 20-µl final volume containing 10 µl of REDExtracti-N-Amp PCR ReadyMix (Sigma-Aldrich, ref. R4775), which includes all the reagents needed for PCR amplification. For the *Cpt1a flox* gene, the *Cpt1a* HomArm forward and reverse primers were used (Additional file 7: Table S1). The DNA fragments obtained were 990 bp for the WT *Cpt1a* amplified allele, 1030 bp for the *Cpt1a* flox allele and 219 bp for the recombined genomic DNA. For the AgRP^CreERT2^ gene, the *AgRP CRE-ER*^*T2*^ forward, reverse and control primers were used (Additional file 7: Table S1). The obtained amplicon was 514 bp from the WT mice and 323 bp for the AgRP^CreERT2^ allele. For the *RiboTag* gene, the *RiboTag loxP* forward and reverse primers were used (Additional file 7: Table S1). The obtained amplicon was 260 bp from the WT mice and 290 bp for the RiboTag allele. For the *ZsGreen* gene, the ZsGreen forward and reverse primers were used (Additional file 7: Table S1). The amplicon obtained was 600 bp from the WT mice and 500 bp for the *ZsGreen* allele.

### Food intake and body weight analysis

All mice had ad libitum food supply and after the recombination the food was monitored weekly during the first month. The initial amount of compound pelleted fodder was weighed at the beginning of every week, using the same precision scale. At the following week, remaining pellets were measured again with the same scale, and food was added to give the same amount as in the previous week. The body weight was monitored weekly until killing using every week the same precision scale.

### Fasting–refeeding satiety test

This test was assessed in 12-week-old mice. Mice were housed in individual cages 2 days before the beginning of the experiment. Mice were fasted overnight (ON) for 12 h and then re-fed a pre-weighed meal. Food intake was measured at 30 min, 1 h, 2 h, 3 h and 4 h after refeeding. All measurements were weighed using the same precision scale [43].

### Ghrelin-induced food intake test

Mice at 12 weeks old were housed in individual cages two days before the beginning of the experiment. Mice were deprived of food for 2 h after the dark period and i.p. injected with ghrelin (0.4 μg/g of body weight; Merck Millipore, Cat# 494127-100G) in physiological saline solution (B. Braun; Cat# 12260029_1019) at 0 min and again at 30 min. We monitored the eating time and food intake for 1 h after the first injection [44].

### Glucose and insulin tolerance tests

For the glucose tolerance test (GTT), mice at 14 weeks old were fasted ON. The baseline blood glucose concentration from a tail snip was measured using a hand glucometer (Bayer, Contour XT, Cat# 83415194) and test strips (Bayer, Contour next, Cat# 84191389). A blood sample was collected in a capillary tube (Deltalab, Cat#7301) and kept in serum separator tubes (Sarstedt, Cat# 201.280) at 4 °C to measure the insulin level. Samples were allowed to clot on ice for 15 min and then centrifuged for 15 min at 5700 rpm. The supernatant was collected and frozen at − 20 °C. Mice were injected i.p. with 20% glucose (Baxter, Cat# 2B0124P) at 1.5 mg/g of body weight. Blood glucose concentrations were measured at 0, 15, 30, 60, 90 and 120 min after the glucose injection. For the insulin tolerance test (ITT), mice were fasted for 6 h. Mice were i.p. injected with 1.5 U of insulin/kg of body weight. Blood glucose concentrations were then measured at 0, 15, 30, 60, 90 and 120 min after the insulin injection.

### Indirect calorimetry

Mice at 12 weeks old were analyzed for EE, respiratory quotient (RQ) (VCO_2_/VO_2_) and locomotor activity (LA) using a calorimetric system (LabMaster; TSE Systems; Bad Homburg, Germany). Briefly, mice were placed in a temperature-controlled (24 °C) cage with flowing air for 48 h for acclimation before starting the measurements. After calibrating the system with the reference gases (20.9% O_2_, 0.05% CO_2_ and 79.05% N_2_), the metabolic rate was measured for 3 days. After that, we extended the experiment for 12 h under fasting conditions and then 4 h under refeeding conditions. O_2_ consumption and CO_2_ production were recorded every 30 min to indirectly determine the RQ. EE, RQ, food intake and LA were measured during the dark and light phases. The LA was assessed using a multidimensional infrared light beam system with the parameters defined by the LabMaster system.

### Non-invasive measurement of blood pressure

Systolic, diastolic and mean blood pressures were measured in conscious mice using the tail-cuff plethysmography method (CODA® tail-cuff blood pressure system; Kent Scientific Corporation; Torrington, CT, USA). 12-week-old mice were trained for tail-cuff measurements over a period of 4 days. Blood pressure measurements were performed at the same time (between 9 a.m. and 12 a.m.) to avoid the influence of the circadian cycle. Blood pressure values were taken from at least ten consecutive measurements [45].

Heat production was visualized using a high-resolution infrared camera (FLIR T420; FLIR Systems, AB, Sweden). Infrared thermography images were taken from the upper half of the body to specifically analyze BAT activity. On the day before the experiment, mice were fasted ON and shaved in the interscapular area to minimize interference. Interscapular BAT temperature was analyzed within a fixed area (region of interest; ROI) using the Flir Tools software (version 4.1). For each image, the average temperature of the skin area was calculated as the average of 3 images/animal.

### Blood and urine collection

Mice were individually placed in metabolic cages that had a floor area of 370 cm^2^ and a length of 207 cm, a width of 267 cm and a height of 140 cm. The cages were adapted with a grid at the bottom to collect urine. 13-week-old mice were housed one day before the start of the experiment, with ad libitum access to food and water. On the day of the study, drinking water and urine volumes were measured for 24 h and urine specimens were taken for osmolality analysis. At the end of the experiment, blood samples were collected in heparinized tubes through the facial vein method [46]. Plasma was collected and frozen at − 20 °C until analysis of the plasma osmolality. At the end of the study, all mice were rehoused in their original cages. One week later, the mice were placed back in the metabolic cages to perform the same protocol under water restriction for 24 h. Urine volume was measured, and blood and urine osmolality was also analyzed. Water intake and urine volumes are expressed as values per gram of body weight.

### Stereotaxic procedure

Mice at 8 week old were anesthetized using 0.1 mg/g of ketamine (Richter Pharma Ag., Cat# 580393.7) and 0.01 mg/g of xylazine (Bayer, Cat#580393.7) before being placed in a stereotaxic apparatus (Kopf instrument, Model 900 Small Animal Stereotactic Instrument). Once the head was shaved with an electric trimmer, a sagittal incision was made through the skin along the midline of the head and a hole was drilled into the skull 1.5 mm posterior and 0.3 mm lateral (left and right) to the bregma (bregma point was found in the perpendicular intersection between the sagittal and coronal synarthroses). A 400 nl of solution containing either AAV9*-EF1a-DIO-mCherry* or AAV1*-EF1a-DIO-synaptophysin-GFP* were injected 50 nl/min using a Hamilton Neuros syringe (5 µl, Neuros Model 75 RN, point style 3, SYR, Cat# 65460-02) and a microinjection pump over 8 min into the ARC. The coordinates for the ARC were − 1.5 mm posterior, ± 0.3 mm lateral and − 5.8 mm ventral to the bregma [47]. Once the procedure was completed, a tissue adhesive (3 M Vetbond™, Cat# 1469Sb) was used to close the incision. Mice were caged with ad libitum access to food and post-surgical drug-supplemented water containing 10% enrofloxacin (Bayer, Cat# 572126.2) and 0.3 g/400 ml of buprenorphine (Indivior, Cat# 679588) as the antibiotic and analgesic, respectively. Daily monitoring was used to follow up on the general state of the operated mice for one week. Three weeks after the adeno-associated viruses (AAVs*)* injection, mice were treated with tamoxifen to induce the recombination.

### Tissue collection

To analyze gene expression, blood metabolites, protein levels and the histological morphology of different tissues, mice were fasted ON and anesthetized with 4% isoflurane (Piramal Healthcare, Cat# 60307-120-25) before being maintained at a surgical plane of anesthesia by the continuous inhalation of 2% isoflurane using a calibrated anesthetic delivery machine (Combi-Vet® Rothacher Medical, Switzerland). Blood was rapidly collected in heparinized tubes (Fibrilin, Cat# 0318) from the descending aorta using a 25-gauge needle (BD Microlance™ 3, Cat# 300600). It was allowed to clot on ice for 15 min and then centrifuged for 15 min at 5700 rpm at 4 °C. The supernatant was collected and frozen at − 20 °C. Samples of liver, inguinal white adipose tissue (iWAT), gonadal white adipose tissue (gWAT), BAT, adrenal gland (AG), pancreas, testis, ovary, kidney, hypothalamus, cortex and hippocampus were collected and stored immediately at − 80 °C until processing. A piece of the tissues was fixed in formalin solution, neutral buffered, 10% (Sigma, Cat# HT501128-4L) for 24 h and then transferred to 1X phosphate buffered saline (PBS) (Sigma, Cat# D1408-500ML) for histological analysis.

To obtain ARC samples, the brain was gently placed in a mouse brain matrix (Agnthos, Cat# 69-2175-1), which can be used to obtain coronal sections with a width starting from 1 mm. A 3-mm coronal section encompassing most of the ARC was obtained, taking as a reference the optical chiasm to establish them for dissection. Once the section was cut, the remaining brain was carefully removed from the matrix and the section was extracted and horizontally positioned. The area was dissected with a crosswise cut starting from the 3rd ventricle up to the base of the hypothalamus. The tissue was immediately stored at − 80 °C until processing.

To perform immunofluorescence assays, whole animal perfusion fixation was applied. Briefly, animals were anesthetized with an i.p. injection of ketamine (Richter Pharma Ag., Cat# 580393.7) and xylazine (Bayer, Cat#580393.7). The delivery dose was 0.1 mg/g of ketamine and 0.01 mg/g of xylazine. Once the animal reached the surgical plane of anesthesia, a 25-gauge blunt perfusion needle connected to a perfusion pump (Gilson, miniplus 3) was inserted into the left ventricle. An incision into the right atrium was performed to create as large an outlet as possible. 75 ml of cold 1X PBS were perfused to remove red blood cells. The PBS was then replaced with 50 ml of 4% paraformaldehyde (PFA), pH 7.4 (Sigma-Aldrich, Cat# 158127). Once the perfusion was complete, the brain was extracted from the skull and fixed in 4% PFA for 4 h at 4 °C. Brains were kept in 30% sucrose (Panreach Applichem, Cat#131621) until they sank. Then, they were frozen in dry ice using pre-cooled 2-methylbutane (Merck, Cat# 277258) and stored at − 80 °C.

To isolate the mRNA from AgRP neurons in vivo, RiboTag-*Cpt1a*KO and control mice were fasted ON and killed by cervical dislocation. The brain was removed from the skull and gently placed in the mouse brain matrix. A 3-mm coronal section containing the ARC was obtained. The section was extracted and horizontally positioned, and the ARC was extracted with punches from the base of the hypothalamus. The tissue was immediately stored at − 80 °C until processing.

## Purification of genomic DNA

Genomic DNA from the different tissues was extracted using the proteinase K method. Briefly, 2.5 µl of proteinase K (0.0001 ng/µl in the reaction) (Thermo Fisher Scientific, Cat# AM2546) were used and the tissue was incubated in 500 µl of lysis buffer (12.2 g of Tris, 1.9 g of EDTA, 2 g of SDS and 11.7 g of NaCl made up to a 1-L solution, pH 8.5) at 55 °C for 4 h. Once digested, 10 µl of RNase A (10 mg/ml) (Sigma-Aldrich, Cat# 10109142001) were added to each tube and incubated at 37 °C for 1 h for RNA degradation. DNA was extracted with 700 µl of phenol:chloroform:isoamyl alcohol (25:24:1) (Merck, Cat# P3803-100ML) and precipitated by 700 µl of 2-propanol (Sigma-Aldrich, Cat# 190764-1L) and 15 µl of 5 M NaCl (Sigma-Aldrich, Cat# S7653-5 KG). The DNA pellet was washed by ethanol (70%) (Panreac, Cat# 361086.1611). The genomic DNA pellet was resuspended in 100 µl of a 10-mM Tris–EDTA solution and stored at 4 °C at least ON before any further processing. The genomic DNA yield was quantified using a NanoDrop 1000 spectrophotometer (Thermo Fisher Scientific, ref. ND-1000).

### Total RNA extraction, cDNA synthesis and qRT-PCR

Depending on the sample, RNA was extracted using the Trizol reagent (Sigma-Aldrich, Cat# T9424) or with a specific kit. For fatty tissues or tissues with a high lipid content, RNA was extracted using the RNeasy Lipid Tissue Mini Kit (QIAGEN, #74804), following the manufacturer's instructions. For the other tissues, the Trizol reagent was used according to the manufacturer’s protocol. RNA samples were heated to 55–60 °C for 10–15 min using a thermoblock (JP Selecta EN, Temblock, Cat# 7462200), quantified using a NanoDrop ND-1000 spectrophotometer and stored at − 80 °C until processing. RNA was reverse transcribed into complementary DNA (cDNA) using TaqMan® Reverse Transcription reagents (Thermo Fisher Scientific, Cat# N808-0234), following the manufacturer’s instructions. The cDNA obtained was diluted in RNase-free water up to a concentration of 10 ng/µl.

Quantitative real-time polymerase chain reaction (qRT-PCR) was performed using the Power SYBR Green PCR Master Mix adapted for the LightCycler 480 system (Roche, Cat# 4887352001), according to the manufacturer’s indication for the LightCycler 480 instrument II (Roche, Cat# 05015243001). mRNA levels from liver tissues were normalized against the β-actin level, while those from BAT and WAT were normalized against the hypoxanthine–guanine phosphoribosyl-transferase (*Hprt*) or β-actin level. Finally, for the cDNA extracted from AgRP neurons, the housekeeping gene used was glyceraldehyde-3-phosphate dehydrogenase (*Gapdh*). All the forward and reverse primers used are described in Additional file 7: Table S1.

### Protein extracts and western blot analysis

Tissues were disrupted by adding 1 ml or 500 µl of a protein extraction buffer to 30–70 mg of non-fat tissues or 50–100 mg of fat tissues, respectively. The protein extraction buffer contained 30 mM HEPES, pH 7.4, 150 mM NaCl, 10% glycerol, 1% Triton X-100, 0.5% sodium deoxycholate (DOC), a Mini Protease Inhibitor Tablet (Roche, Cat# 11836153001) and a PhosSTOP Phosphatase Inhibitor Tablet (Roche, Cat# 04906837001). To disrupt the tissue, the TissueLyser LT was used for 3 min at 50 Hz. Protein concentration of the samples were quantified using the Pierce™ BCA Protein Assay Kit (Thermo Fisher Scientific, Cat# 23227), following the manufacturer’s instructions. Protein lysates were separated in SDS-PAGE and transferred on to 0.45-µm nitrocellulose membranes (Bio-Rad Laboratories, Cat# 1620115), which were incubated ON with primary antibodies (Additional file 7: Table S2). Images were acquired with an ImageQuant LAS 4000 mini developer system (GE Healthcare Life Sciences). The images obtained were processed with Fiji ImageJ1.33 software (NIH; Bethesda, MD, USA) to quantify the average optical density of all the immunoreactive bands.

### Histopathology

Fixed tissues were dehydrated and paraffin-embedded. The resulting blocks were cut into 4-µm sections and stained with hematoxylin and eosin (H&E) to assess histology. Photomicrographs were obtained using a microscope camera (Leica MC 190 HD Camera) and microscope (Leica DM IL LED Tissue Culture Microscope). The images shown are representative of 10 biological replicates per condition.

### Assessment of urine and serum osmolality

Urine osmolarity (U_osm_) and serum osmolality (P_osm_) were measured with 20-µl samples using the freezing-point depression technique involving an osmometer (3320 Micro-Osmometer, Advanced Instruments). A control (Clinitrol 290) and a set of calibration standards (50, 850 and 2000 mosm/kg H_2_O) were used before running each batch.

### Plasma and urine analyses

Plasma insulin (Insulin ELISA Kit, Alpco, Cat# 80-INSHU-E01.1), aldosterone (Aldosterone ELISA Kit, LSBio, Cat# LS-F28206-1), VP/ADH (Vasopressin ADH ELISA Kit, LSBio, Cat# LS-F7592-1), renin (Renin ELISA kit, Elabscience, Cat# E-EL-M0061), angiotensin (Angiotensin ELISA Kit, LSBio, Cat# LS-F67331-1), leptin (Leptin ELISA kit, Sigma-Aldrich, Cat# RAB0334), 17-β-estradiol (Estradiol ELISA kit, Cayman, Cat# 501890), testosterone (Testosterone ELISA kit, Arbor Assays, Cat# K080-H1), glucose (Monlab, Cat# MO-165086), TGs and non-esterified fatty acids (NEFAs) (NEFA-HR kit, Wako, Cat#434-91795, 436-91995, 270-77000) levels were all measured according to the manufacturer’s instructions.

### Immunohistochemistry

To determine the tissue distribution of the protein of interest, immunofluorescence for free-floating brain sections was performed. Frozen brains were embedded in OCT (Tissue-Tek, Cat#4583) and 30-μm slices were obtained with a microtome (Leica SM2000R). Tissue slices were stored at 4 °C in a cryoprotectant solution [30% (w/v) sucrose, 30% (v/v) ethylene glycol and 250 ml of PBS] until the immunostaining was performed. Tissue slices were washed 3 times for 5 min in 1X PBS to remove the cryoprotectant solution. All steps were performed under gentle agitation in a shaker (Thermo-Shaker, PST-100HL). Slices were permeabilized in potassium phosphate buffered saline (KPBS) [0.9% (p/v) NaCl, 52 mM potassium phosphate dibasic and 9.6 mM potassium dihydrogen phosphate] containing 0.1% Triton X-100, for 10 min and blocked for 1 h with blocking solution (KPBS containing 0.1% (v/v) Triton X-100, 3% (w/v) BSA and 2% (v/v) goat serum (Sigma-Aldrich, Cat# G9023). The slices were incubated with the primary antibody in blocking solution for 1 h at room temperature and ON at 4 °C. They were then washed 3 times for 10 min with KPBS containing 0.1% Triton X-100 before being incubated with the secondary antibody in blocking solution for 2 h at room temperature and protected from the light. Finally, the samples were washed 3 times for 10 min with KPBS containing 0.1% Triton X-100 and mounted onto SuperFrost Plus slides (Thermo Fisher Scientific, Cat# J7800AMNT) with fluoromount-G containing DAPI (Thermo Fisher Scientific, Cat# 00-4959-52) and cover slipped. The primary and secondary antibodies are listed in Additional file 7: Table S2 showing the dilutions used in each incubation. Fos-positive cells and synaptophysin fluorescence in PVN sections were quantified using the Fiji ImageJ 1.33 (NIH; Bethesda, MD, USA), consistent with previous studies [48].

### Dendritic spine analysis

For detailed morphological analyses of dendritic spines, samples were imaged with a Zeiss LSM 800 confocal microscope using a 1003 Plan Apo TIRF DIC-oil immersion objective (total magnification of 63×). To visualize the mCherry protein in AgRP neurons, samples were excited with a 587-nm laser and the fluorophore emission was captured by a 610 band-pass filter. A Z-stack was obtained for each dendrite extending from the apex of the cell soma. For each animal, 20 dendrites (segmented by 50 µm) were analyzed. Z-stacks were used for three-dimensional reconstruction. The dendritic spine density in each dendritic segment was quantified using the Fiji ImageJ 1.33 (NIH; Bethesda, MD, USA) and is expressed as the dendrite number/50 µm.

### Adipocyte area measurement

To quantify the adipocyte area, three representative images from each adipose tissue section were taken with a 20× objective using a high-sensitivity camera (Leica MC 190 HD Camera). Images were analyzed with the Adiposoft software, a fully automated open-source program for the analysis of WAT cellularity in histological sections [49]. Each image was calibrated to 4.65 pixels per micron using a 20× objective and a Leica microscope. Any adipocyte with visible alterations in the membranes was “closed” digitally prior to continuing with the automated quantification. To quantify the adipocyte area, images were analyzed and adipocytes were highlighted if they met the following criteria: (1) the boundaries for sizing of the cell were 40–40,000; (2) the adipocyte had a shape factor of 0.35–1 (a shape factor of 0 indicated a straight line, while a shape factor of 1 indicated a perfect circle); and (3) the adipocyte did not border the image frame. The results are represented as the frequency distribution and the average of total area counted.

### Mitochondrial content

Sections containing the ARC from ZsGreen mice at 12 weeks old fasted ON were scanned with (Multiphoton Microscope Leica TCS SP8 MP). The LAXZ software was used to obtain high-quality images. Images were acquired sequentially, using 405- and 488-nm laser lines, with a 63× oil immersion objective. The confocal pinhole was set at 1 Airy unit. The format was 1024 × 1024 pixel. Specific settings for the frame averaging (6 frames per image) and laser gain (900 for the 488-nm laser and 800 for the 405-nm laser) were selected for all the images to improve quality. For analysis, Z-stacks over the diameter of AgRP neurons were performed with a zoom of 4×. The analysis was performed using the Fiji ImageJ1.33 software (NIH; Bethesda, MD, USA).

### Transcriptomic analysis with the RiboTag technique

Punches containing the ARC from mice at 12 weeks old fasted ON were homogenized in 350 µl of homogenization buffer (50 mM Tris, 100 mM KCl, 12 mM MgCl2, 1% Nonidet P-40, 1 mM DTT, 200 U/ml Promega RNasin, 1 mg/ml heparin, 100 μg/ml cycloheximide, Sigma protease inhibitor mixture at pH 7.5) with a 30G needle. After clearing, 30 μl was separated as input and stored at − 80 °C until further processing. 3 µl of the anti-HA antibody (BioLegend, Cat# MMS-101R) were added to the remaining supernatant and samples were incubated in a cold-room spinner for 2 h. After incubation, 200 μl of Dynabeads protein G magnetic beads (Thermo Fisher Scientific, Cat# 10004D) were added and incubated for 2 h at 4 °C with rotation. Next, beads were washed 3 times for 10 min with gentle rotation at 4 °C in high-salt buffer (50 mM Tris, 300 mM KCl, 12 mM MgCl2, 1% Nonidet P-40, 1 mM DTT, 100 μg/ml cycloheximide at pH 7.5). After the final wash, beads were incubated with 350 µl of the RLT buffer (QIAGEN, Cat# 74034). Total RNA was prepared according to the manufacturer’s instructions using the RNeasy-plus Mini kit (QIAGEN, Cat# 74034). RNA was quantified with the Quant-iT RiboGreen RNA (Thermo Fisher Scientific, Cat# R11490). cDNA was generated using the SuperScript IV reverse transcriptase (Thermo Fisher Scientific, Cat# 11750150) following the manufacturer’s instructions. Briefly, 0.27 ng of RNA from the samples and the input was incubated with 5X SuperScript IV RT buffer, 100 mM DTT, the RNaseOUT Recombinant RNase inhibitor and the SuperScript IV reverse transcriptase (200 U/µl). The samples were incubated at 23 °C for 10 min, 50 °C for 10 min and 80 °C for 10 min. They were then stored at − 20 °C until the qRT-PCR was performed. qRT-PCR was performed with the Taqman probes for *Agouti-related peptide* (Agrp): Mm00475829_g1; *aldehyde dehydrogenase 1 family member L1 (Aldh1/1)*: Mm03048957_m1; *Discs large MAGUK scaffold protein 4*(*Dlg4*): Mm00492193_m1; *Glutamic acid decarboxylase 1* (*Gad1*): Mm04207432_g1; *Neuropeptide y* (*Npy*): 00445771m1; *Proopiomelanocortin* (Pomc): Mm00435874_m1; *Solute carrier family 32 member 1* (*Slc32a1*): Mm00494138_m1; *Solute carrier family 17 member 6* also called *Vesicular glutamate transporter 2* (*Slc17a6*): Mm 00499876m1 (Thermo Fisher Scientific).

### Quantification and statistical analysis

Statistical significance was determined using the Prism 8.0 software from GraphPad (GraphPad Software, La Jolla, CA, USA). Two-way ANOVA followed by post hoc analysis with the Sidak and Bonferroni test in the multiple comparison analysis were applied when more than two groups were compared. Student’s t-test was performed when two groups were compared. Data are expressed as the mean ± SEM. A p value lower than 0.05 was considered significant. Fiji ImageJ 1.33 (NIH; Bethesda, MD, USA) was used to determine the levels of Fos-positive cells, GFP-syn, mitochondria and dendritic spines as well as for western blot analyses. The numbers of animals used in each experimental setting and analysis are specified in each figure legend.

## Results

### CPT1A in AgRP neurons is involved in energy balance sexual-dimorphic phenotype

We generated mice with a selective deletion of *Cpt1a* in their AgRP neurons at adult stage (*Cpt1a*KO mice). Mice expressing tamoxifen-inducible Cre recombinase (*CreERT2*) in their AgRP neurons (AgRP^CreERT2^*)* [41] were crossed with mice harboring conditional alleles of *Cpt1a* (*Cpt1a*^flox/flox^ mice) obtained in our laboratory [40]. Tamoxifen injections were done at 8-week-old mice and they were all studied 4 weeks after the injection. To confirm Cre expression in the AgRP neurons, we injected *AAV9-EF1a-DIO-mCherry* into the ARC in *Cpt1a*KO mice and we observed that AgRP neurons showed red fluorescence (Additional file 1: Fig. S1a–c). To corroborate the deletion of *Cpt1a* exon 4 in the AgRP neurons we isolated genomic DNA from the ARC and several other tissues from both *Cpt1a*KO and control mice. In order to distinguish the recombined *Cpt1a* gene lacking exon 4 from WT *Cpt1a* gene, which is also expressed in other cell types, we firstly digested the genomic DNA from all studied tissues with the restriction enzymes *Pst*I and *Aat*II. The restriction sites in those two enzymes are only present in the WT *Cpt1a* gene but not in the recombined *Cpt1a* gene lacking exon 4 (Additional file 1: Fig. S1d, e). Next, we confirmed the *Cpt1a* recombination by using PCR amplification of the loxP-flanked region containing *Cpt1a* exon 4. The resulting recombined *Cpt1a* amplification band was 219 bp and it was only detected in the genomic DNA from ARC. This amplicon was also sequenced and our results confirmed that *Cpt1a* exon 4 was only removed in the genomic DNA from ARC of *Cpt1a*KO mice (Additional file 1: Fig. S1g). The deletion of exon 4 has been shown previously by our group to result in a reading frame change and an appearance of a stop codon in exon 5 of the recombined *Cpt1a* gene [40]. Since AgRP is also expressed by chromaffin cells of the adrenal medulla [50], we analyzed adrenal gland histology, weight, *Tyrosine hydroxylase (Th)* mRNA levels (indicative of adrenal gland integrity). No changes in histology, adrenal gland weight and *Th* mRNA levels were observed in both female and male *Cpt1a*KO mice with respect to their control littermates (Additional file 2: Fig. S2a–e).

**Fig. 1:**
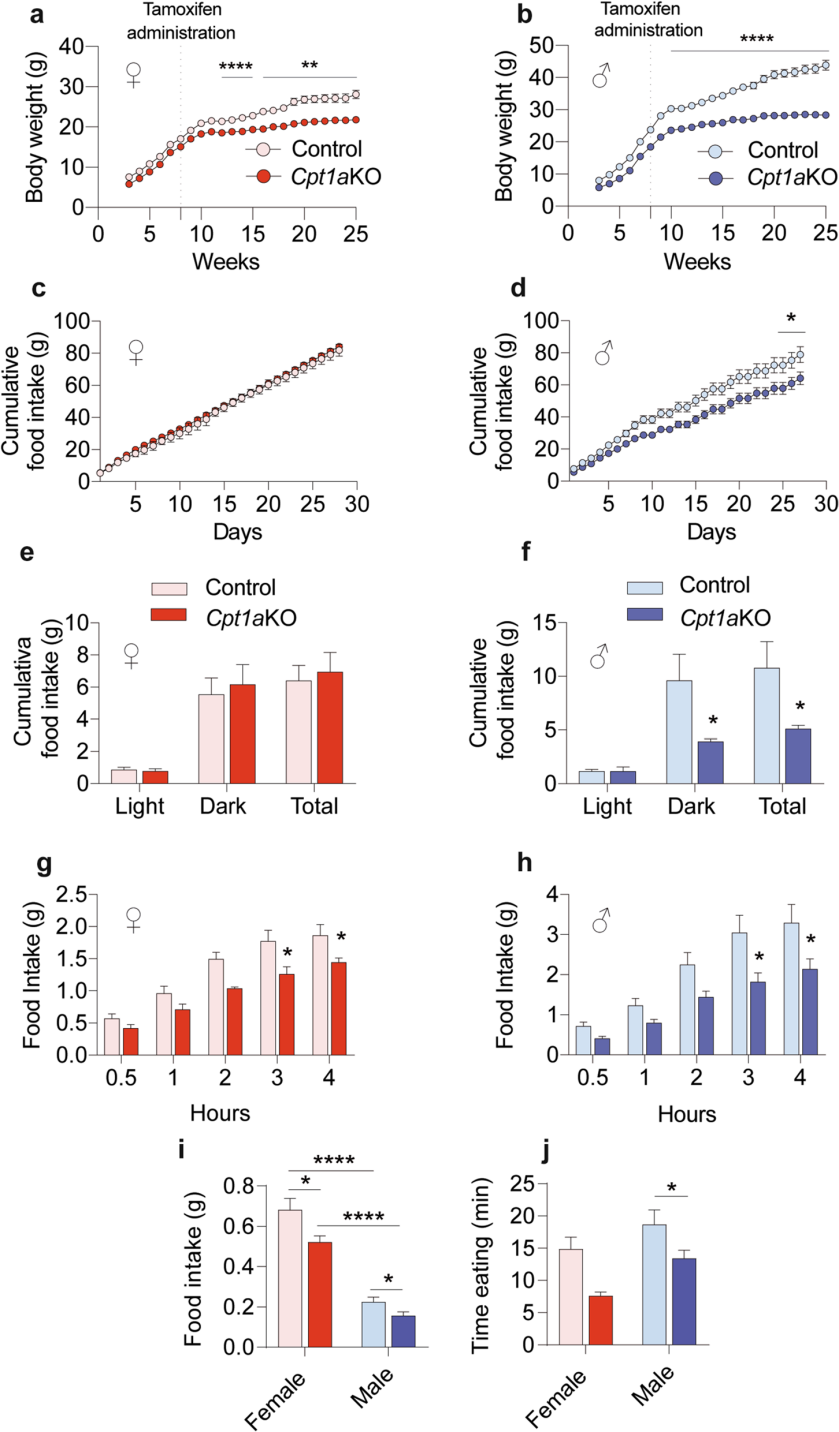
Deletion of *Cpt1a* in AgRP neurons affects feeding behavior in a sex-dependent manner. **a**, **b** Body weight in *Cpt1a*KO female in red (**a**, *n* = 8) and male mice in blue (**b**, *n* = 6) vs their control littermates (female *n* = 10, and male *n* = 9). **c**, **d** Cumulative food intake in female (**c**, *n* = 8–10) and male (**d**, *n* = 6–9) mice measured during 1 month after tamoxifen induction. **e**, **f** Analysis of food intake measured by the TSE system during light and dark phases in female (**e**, *n* = 6–6) and male mice during 3 consecutive days (**f**, *n* = 6–6). **g**, **h** Response to fasting–refeeding in female (**g**, *n* = 8–10) and male mice (**h**, *n* = 10–8). **(i)** Cumulative food intake measured 1 h after intraperitoneal (i.p.) ghrelin administration in female (*n* = 7–7) and male mice (*n* = 6–6). **j** Time that female (*n* = 7–7) and male mice (*n* = 6–6) spent eating after the ghrelin injection. Data are expressed as the mean ± SEM. In a–d, *p < 0.05, **p < 0.01, ****p < 0.0001, using two-way repeated-measures ANOVA followed by Šidák’s post hoc test. In **e** and **f**, * *p* < 0.05, using Student’s *t*-test. In **g** and **h**, *p < 0.05 using two-way repeated-measures ANOVA followed by Šidák’s post hoc test. In **i**–**j**, *p < 0.05, **p < 0.01, using Student’s t-test. In **i**–**j** *p < 0.05, ****p < 0.0001, using two-way ANOVA followed by Turkey post hoc test

When we assessed the study of the phenotype we observed that female and male *Cpt1a*KO mice gained less weight than their control littermates when fed a normal chow diet ad libitum (Fig. 1a, b and Additional file 3: Fig. S3a). However, only the males showed a reduction in food intake (Fig. 1c, d and Additional file 1: Fig. S3b, c). This was confirmed by the food intake analysis during the dark phase (Fig. 1e and f), indicating sex differences in the function of AgRP neurons. To gain more insight into this difference in food intake behavior, we analyzed feeding patterns after an ON fast or after the i.p. ghrelin administration. Both female and male *Cpt1a*KO mice showed an impaired feeding response after an ON fast compared to the control mice (Fig. 1g and h). However, the food intake reduction was greater in male *Cpt1a*KO mice. Interestingly, ghrelin administration induced greater food ingestion in control than in *Cpt1a*KO female mice. This was also observed in male mice. However, the food intake observed in the females was 3 times greater than that observed in the males, indicating that the females were more sensitive to ghrelin than the males (Fig. 1i). Consistent with these results, male *Cpt1a*KO mice spent less time eating food than the female mice (Fig. 1j). Altogether, these results suggested a different role of CPT1A in males and females in the AgRP neurons under ad libitum conditions and after ghrelin administration.

### Cpt1a deletion in AgRP neurons increases energy expenditure (EE)

**Fig. 2:**
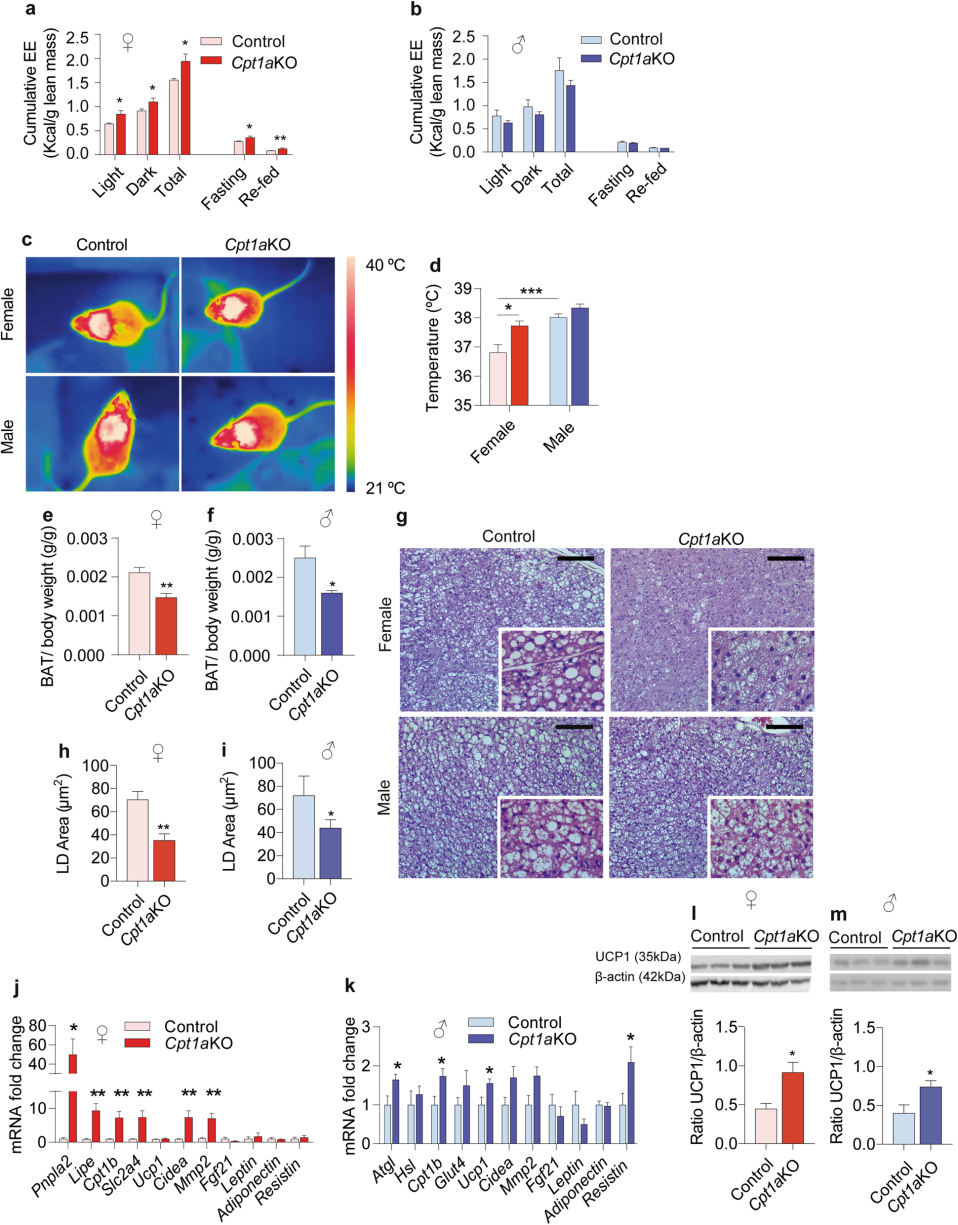
*Cpt1a* ablation in AgRP neurons increases brown adipose tissue activity. **a**, **b** EE profile normalized against the lean body mass in female (**a**, *n* = 6–6) and male mice (**b**, *n* = 6–6). **c** Representative infrared thermal images of female (top panel, *n* = 11–9) and male mice (bottom panel, *n* = 8–7). **d** Quantification of the interscapular temperature adjacent to the brown adipose tissue (BAT) of female (*n* = 11–9) and male mice (*n* = 8–7). **e**, **f** Weight of BAT normalized against the body weight of female (e, *n* = 6–9) and male mice (**f**, *n* = 6–7). **g** Representative images of BAT sections dyed with the H&E stain in female (top panel) and male mice (bottom panel). Scale bar, 100 μm (×magnification 20). **h**, **i** Lipid droplet quantification using ImageJ in female (**h**, *n* = 4–4) and male mice (**i**, *n* = 4–4). **j**, **k** Analysis by qRT-PCR of the mRNA levels of *Pnpla2, Lipe, Cpt1b, Slc2a4 Ucp1, Cidea,* *Mmp2, Leptin, Adiponectin and Resistin* in female (**j**, *n* = 7–7) and male mice (**k**, *n* = 5–6). **l**, **m** Representative western blot of UCP1 protein levels in BAT (30 μg) from female (**l**, *n* = 6–8) and male mice (**m**, *n* = 6–8) normalized against β-actin levels. Data are expressed as the mean ± SEM. In **d** * *p* < 0.05, ***p < 0.001, using two-way ANOVA followed by Turkey post hoc test. In **a**, **b**, **e**, **f**, **h**, **I**, **j**–**m**, *p < 0.05, **p < 0.01, using Student’s *t*-test

Analysis of metabolic parameters revealed differences in EE between the sexes. *Cpt1a*KO female mice showed increased EE compared to the control female mice. These changes in EE were only observed in *Cpt1a*KO female mice but not in male mice (Fig. 2a and b and Additional file 3: Fig. S3d–f). No changes were observed in the RQ (Additional file 3: Fig. S3g, h) or in locomotor activity (Additional file 3: Fig. S3i, j) in both sexes. However, only *Cpt1a*KO female mice increased their RQ to 0.95 in refeeding conditions after an ON fast (Additional file 3: Fig. S3g).

To determine whether the *Cpt1a* ablation from AgRP neurons enhanced EE in female mice, we measured the interscapular BAT temperature as an indicator of activated BAT thermogenesis. *Cpt1a*KO female mice showed a substantial increase in the BAT temperature compared to the control female mice (Fig. 2c and d). Consistent with this, *Cpt1a*KO female mice showed a significant reduction in BAT weight and lipid droplet (LD) area compared to their control littermates and the male mice (Fig. 2e–i). At the molecular level, the UCP1 protein concentration and mRNA levels of thermogenesis-related genes (*Cidea* and *Mmp2*), lipolytic genes (*Pnpla2* and *Lipe*) and FAO markers (*Cpt1b*) were also elevated, confirming an enhanced activation of thermogenesis in *Cpt1a*KO female mice compared to their control littermates (Fig. 2j). All this thermogenic activity seemed to respond to an activated sympathetic tone. The thermogenic response and LD reduction were much lower in the *Cpt1a*KO male mice than in the *Cpt1a*KO female mice (Fig. 2d, j–m), in accordance with the higher EE observed in the *Cpt1a*KO female mice. In addition, we analyzed the mRNA levels of *leptin, adiponectin and resistin*. We only observed an increase in the *resistin* mRNA levels in *Cpt1a*KO male mice.

**Fig. 3:**
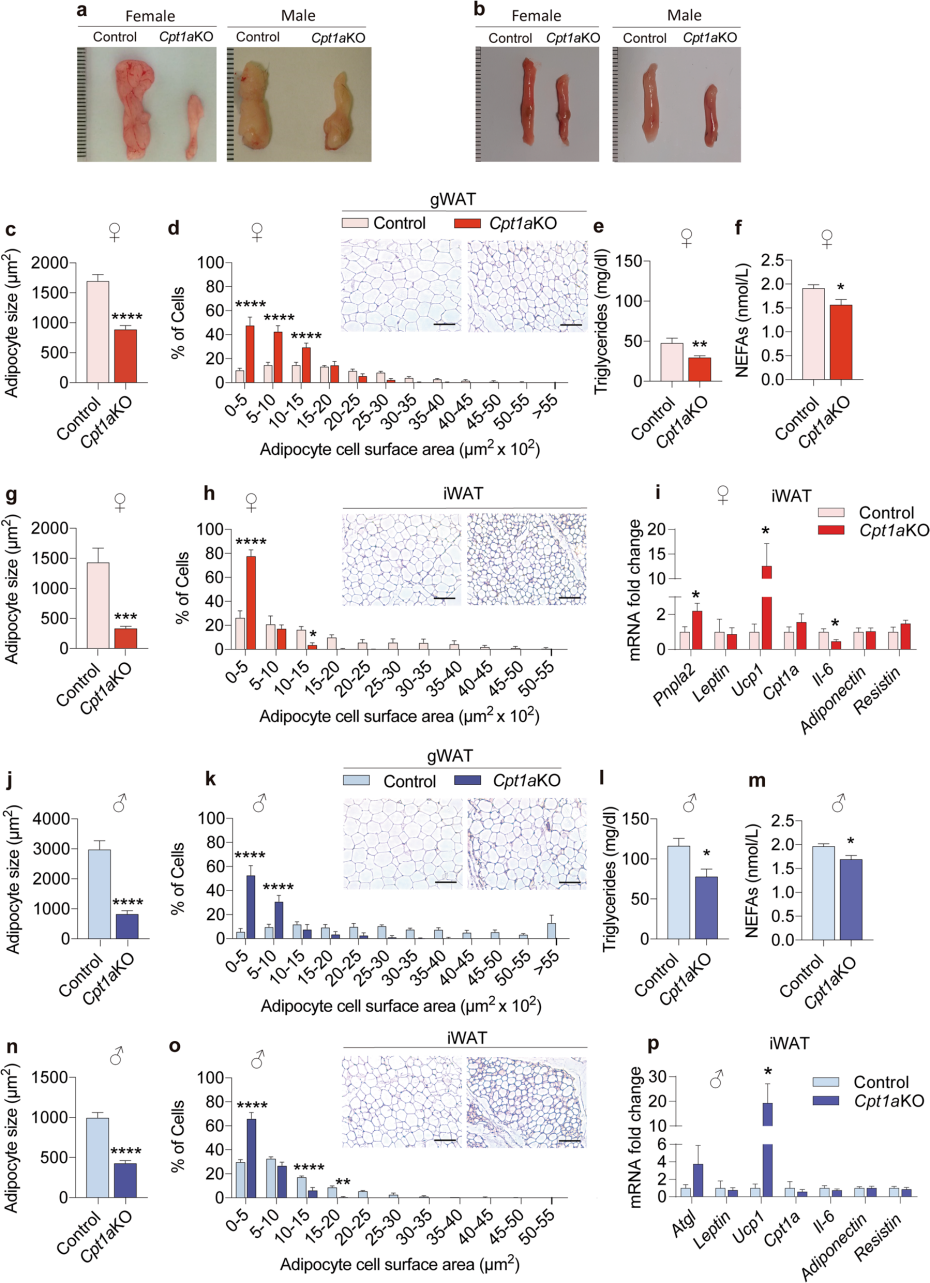
*Cpt1a* ablation in AgRP neurons reduces lipid content in white adipose tissue. **a**, **b** Representative image of gonadal white adipose tissue (gWAT) (**a**) and inguinal white adipose tissue (iWAT) (**b**) of female and male mice vs their control littermates. **c**, **j** Average adipocyte area of gWAT in female (**c**, *n* = 10–10) and male mice (**j**, *n* = 10–15). **d**, **k** Morphometric analysis of the adipocyte area distribution in the gWAT of female (**d**, *n* = 5–5) and male mice (**k**, *n* = 5–5). **g**, **n** Average adipocyte area of iWAT in female (**g**, *n* = 10–10) and male mice (**n**, *n* = 8–8). **h**, **o** Morphometric analysis of adipocyte area distribution in the iWAT of female (**h**, *n* = 5–5) and male mice (**o**, *n* = 5–5). **e**, **f** TG and NEFAs measurement in female (*n* = 5–5) and male mice (*n* = 5–5) (**l**–**m**). **i**, **p** Analysis by qRT-PCR of the mRNA levels of *Pnpla2, Leptin, Ucp1, Cpt1a,* *Il6, Adiponectin and Resistin* in the iWAT of female (**i**, *n* = 8–7) and male mice (**p**, *n* = 6–6). Data are expressed as the mean ± SEM. In **d**, **h**, **k**, **o**, *p < 0.05, **p < 0.001, ****i < 0.0001, using two-way repeated-measures ANOVA followed by Šidák’s post hoc test. In **c**, **e**, **f**, **g**, **i**, **j**, **l**, **m**, **n**, **p**, *p < 0.05, **p < 0.01, ***p < 0.001, ****p < 0.0001 using Student’s *t*-test

We also analyzed the effects of *Cpt1a* ablation in AgRP neurons on different tissues. An important reduction was observed in the selected WAT deposits in *Cpt1a*KO mice compared to the control mice (Fig. 3a and b and Additional file 4: Fig. S4a and b), which was consistent with the observed reduction in body weight. Gonadal and inguinal WAT (gWAT and iWAT, respectively) of *Cpt1a*KO mice of both sexes showed a reduced adipocyte size with respect to their control littermates (Fig. 3c, g, j and n). Interestingly, while *Cpt1a*KO female mice showed a major reduction in iWAT (76% iWAT reduction vs 45.5% gWAT reduction), gWAT was the most affected tissue in *Cpt1a*KO male mice (70% gWAT reduction vs 56.7% iWAT reduction) (Fig. 3c, g, j and n). These results were consistent with the analysis of the frequency distribution of the adipocyte area, since a higher frequency of smaller adipocytes (< 500 μm^2^) in the iWAT of *Cpt1a*KO female mice corresponded to 80% of the tissue (Fig. 3g). In addition, we observed an enhanced browning in the iWAT of both sexes (Fig. 3h and o), consistent with the increased *Ucp1* mRNA levels (Fig. 3i and p). We also measured serum leptin levels (Additional file 4: Fig. S4c and S4d) but no significant changes were observed between controls and *Cpt1a*KO mice. Furthermore, we measured serum TG and NEFAs levels in both sexes. A reduction in serum TGs and NEFAs levels was observed in *Cpt1a*KO female and male mice (Fig. 3e, f, l and m). These results are suggestive that deletion of *Cpt1a* from AgRP neurons activated the sympathetic nervous system, leading to enhanced thermogenesis mainly in *Cpt1a*KO female mice, an increased browning of the iWAT in both sexes as well as considerably reduced fat deposits consistent with the reduction in body weight.

We also analyzed other tissues such as the liver, pancreas, testicles, and ovaries. No morphological changes were observed in these tissues (Additional file 5: Fig. S5). Moreover, no changes were observed in the liver weight in both sexes (Additional file 5: Fig. S5b and d). However, when we assessed the mRNA levels of the marker genes involved in glucose and FA metabolism, we observed that *Cpt1a*KO male mice showed increased mRNA levels of the gluconeogenic genes encoding phosphoenolpyruvate carboxykinase (*Pepck*) and glucose 6-phosphatase (*G6pc*) (Additional file 5: Fig. S5c and e). These changes were not observed in the female mice. Expression *Cpt1a* was increased in both sexes, but the expression of *Ucp*2 was increased only in the *Cpt1a*KO male mice. These results suggested a sex-dependent difference in the liver metabolic adaptation during fasting to the deletion of *Cpt1a* in AgRP neurons. Although we did not observe morphological changes in ovaries and testis, we measured the plasma levels of 17-β-estradiol in females and testosterone in males (Additional file 5: Fig. S5g and h). No changes were observed in circulating levels of 17β-estradiol in females. However, we observed a significative reduction of plasma testosterone levels in *Cpt1a*KO male mice.

### *CPT1A* in AgRP neurons is involved in thirst control

**Fig. 4:**
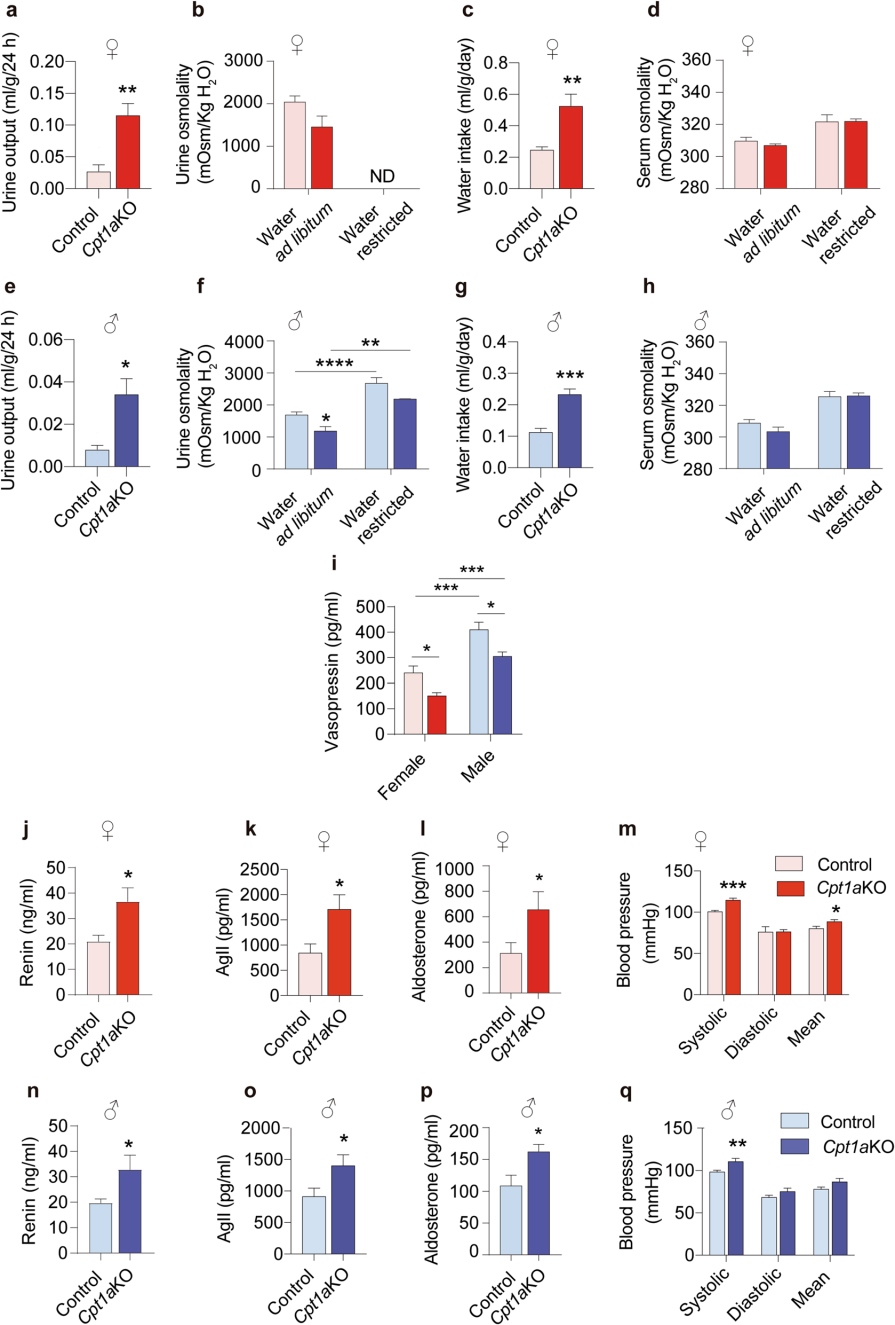
*Cpt1a*KO mice display changes in thirst behavior. **a**, **e** 24 h of urine collection in female (**a**, *n* = 6–5) and male mice (**e**, *n* = 6–7). **b**, **f** Analysis of urine osmolality in female (**b**, *n* = 11–8) and male mice (**f**, *n* = 16–9) under 24 h of water restriction or ad libitum access to water; ND, not detected. **c**, **g** Total amount of water intake for 24 h in female (**c**, *n* = 6–7) and male mice (**g**, *n* = 6–5). **d**, **h** Analysis of serum osmolality in female (d, n = 10–5) and male mice (**h**, *n* = 9–6) under 24 h of water restriction or ad libitum access to water. **i** Plasma level of the vasopressin hormone in female (*n* = 8–8) and male mice (*n* = 9–6). **j**, **n** Plasma levels of renin in female (**j**, *n* = 5) and male mice (**n**, *n* = 7–9). **k**, **o** Plasma levels of angiotensin II in female (**k**, *n* = 10–8) and male mice (**o**, *n* = 9–6). **l**, **p** Plasma levels of aldosterone in female (l, n = 8) and male mice (**p**, *n* = 8–10). **m**, **q** Blood pressure in female (**m**, *n* = 5–5) and male mice (**q**, *n* = 8–8). Data are expressed as the mean ± SEM. In **b**, **d**, **f**, **h**, **i**, **p < 0.01, ****p < 0.0001 using two-way repeated-measures ANOVA followed by the post hoc Bonferroni test. In **a**, **c**, **e**, **g**, **j**–**o**, *p < 0.05, **p < 0.01, ***p < 0.001, using Student’s *t*-test

One week after tamoxifen administration and Cre induction, we observed that female and male *Cpt1a*KO mice resulted in polyuria. Thus, mice were housed in metabolic cages for 24 h to study their water balance. Ad libitum water intake in both female and male *Cpt1a*KO mice led to the excretion of an abnormally large volume of urine each day, which was more prominent in the female mice (Fig. 4a and e). This was accompanied by low urine osmolality, indicating high urine dilution (Fig. 4b and f). As expected, given their polyuria, female and male *Cpt1a*KO mice showed water consumption that was twice that of their control littermates (Fig. 4c and g). Interestingly, no significant changes were observed in serum osmolality, suggesting that the excretion of very diluted urine was compensated by the high intake of water (Fig. 4d and h).

To distinguish the *Cpt1a*KO polyuria from a diabetic condition, GTT and ITT were performed. *Cpt1a*KO male mice showed significantly increased glucose tolerance compared to their control littermates (Additional file 6: Fig. S6f and g). Despite females having a slight change in the first GTT timepoint, the overall glucose sensitivity measured as AUC was not improved (Additional file 6: Fig. S6a, b). No differences were observed in the ITT in both sexes (Additional file 6: Fig. S6c and h). Next, we measured serum insulin levels and urine glucose content. No alterations were observed in these two parameters in the *Cpt1a*KO mice with respect to the control mice (Additional file 6: Fig. S6d, e, i, j). In addition, no changes were observed in the morphology of the kidneys in both sexes, excluding an intrinsic renal disease (Additional file 6: Fig. S6k). These data emphasized that *Cpt1a*KO mice did not suffer type II diabetes, but they did have disrupted water intake.

To further understand the mechanisms that caused polydipsia, mice were deprived of water for 24 h in the metabolic cages. During dehydration, male *Cpt1a*KO mice showed a significantly increased urine osmolality, as well as their control littermates (Fig. 4f). Unfortunately, we were not able to collect enough urine volume to perform this analysis in the female mice under water deprivation (Fig. 4b). Despite this, no differences in serum osmolality were observed in response to water restriction (Fig. 4d and h). These data indicated that *Cpt1a*KO mice partially conserved their ability to respond to water deprivation**.** We then analyzed plasma levels of vasopressin (VP, also known as anti-diuretic hormone, ADH) an important regulator of fluid balance and is synthesized in magnocellular neuronal cell bodies of the PVN and SON [51]. We observed reduced circulating levels of VP/ADH in both sexes. However, the effect was more prominent in female *Cpt1a*KO mice (Fig. 4i). Another critical regulator of fluid balance and blood pressure is the renin–angiotensin–aldosterone system (RAAS). Typically, the RAAS is activated when there is a drop in blood volume to increase water and electrolyte reabsorption in the kidneys. Thus, we determined the levels of renin, angiotensin II (AgII) and aldosterone as important integrators of the RAAS and blood pressure. Both female and male *Cpt1a*KO mice showed increased circulating levels of renin, AgII and aldosterone (Fig. 4j–l and n–p), which were accompanied by a mild increase in the systolic blood pressure (Fig. 4m and q). Taken together, these data suggest that lipid metabolism in AgRP neurons is involved in the regulation of fluid homeostasis.

### CPT1A is required in AgRP neurons for spine and presynaptic terminal formation

**Fig. 5:**
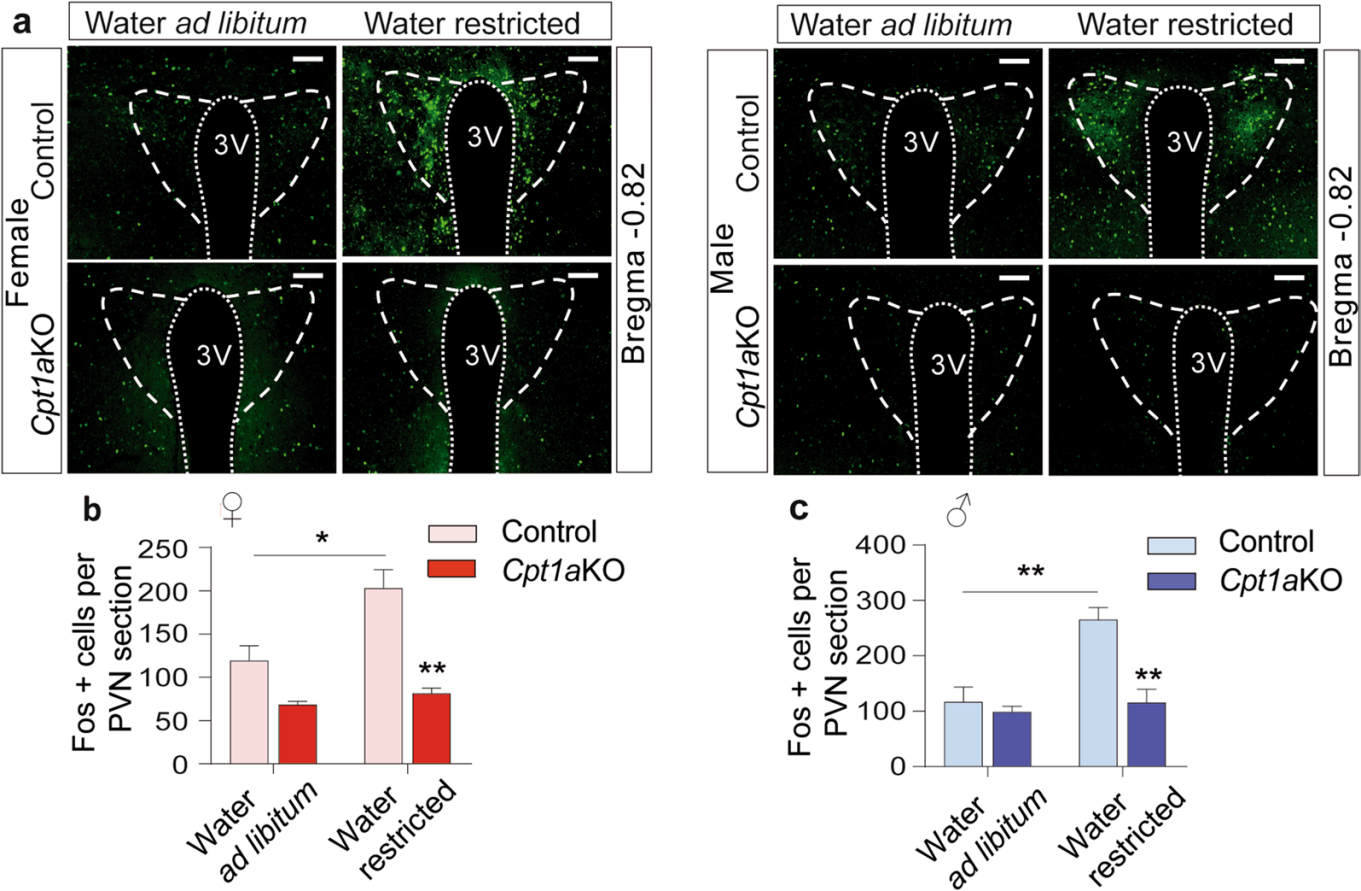
Deletion of *Cpt1a* in AgRP neurons impairs PVN activation during water restriction. **a** Representative images of Fos activation in the paraventricular nucleus (PVN) of female (left panel) and male mice (right panel) under conditions of ad libitum access to water or 24 h of water restriction. Dashed lines indicate the PVN area analyzed. **b**, **c** Quantification of Fos-positive cells per PVN section in female (**b**, *n* = 4–4) and male (**c**, *n* = 4–4) mice. Data are expressed as the mean ± SEM. In **b** and **a**, *p < 0.05, **p < 0.001, using two-way ANOVA followed by the post hoc Bonferroni test

Considering that the PVN is an important target of AgRP neuronal projections and since VP/ADH is released from the axonal projections of paraventricular magnocellular cells into different brain areas (the rostro lateral medulla, nucleus tractus solitarius and intermediolateral column of the spinal cord) that are responsible for the integration of the peripheral sympathetic and vagal outflow [52, 53], we wanted to know whether *Cpt1a* deletion from AgRP neurons affected the activity of PVN neurons. Immunofluorescence assays of Fos were used to detect the pattern of PVN activity under ad libitum access to water or in response to 24 h of water restriction. No statistical differences were observed in Fos activation in the PVN under ad libitum access to water in both female and male mice. This was interesting since male and female *Cpt1a*KO mice showed an increase in water intake with no sign of Fos activation in the PVN during free access to water (Fig. 5a–c). However, we observed a significant reduction in Fos activation in both female and male *Cpt1a*KO mice compared with their control littermates during water deprivation (Fig. 5a–c). Thus, these results suggested that the AgRP neurons in *Cpt1a*KO mice reduced their neuronal connections to the PVN.

**Fig. 6:**
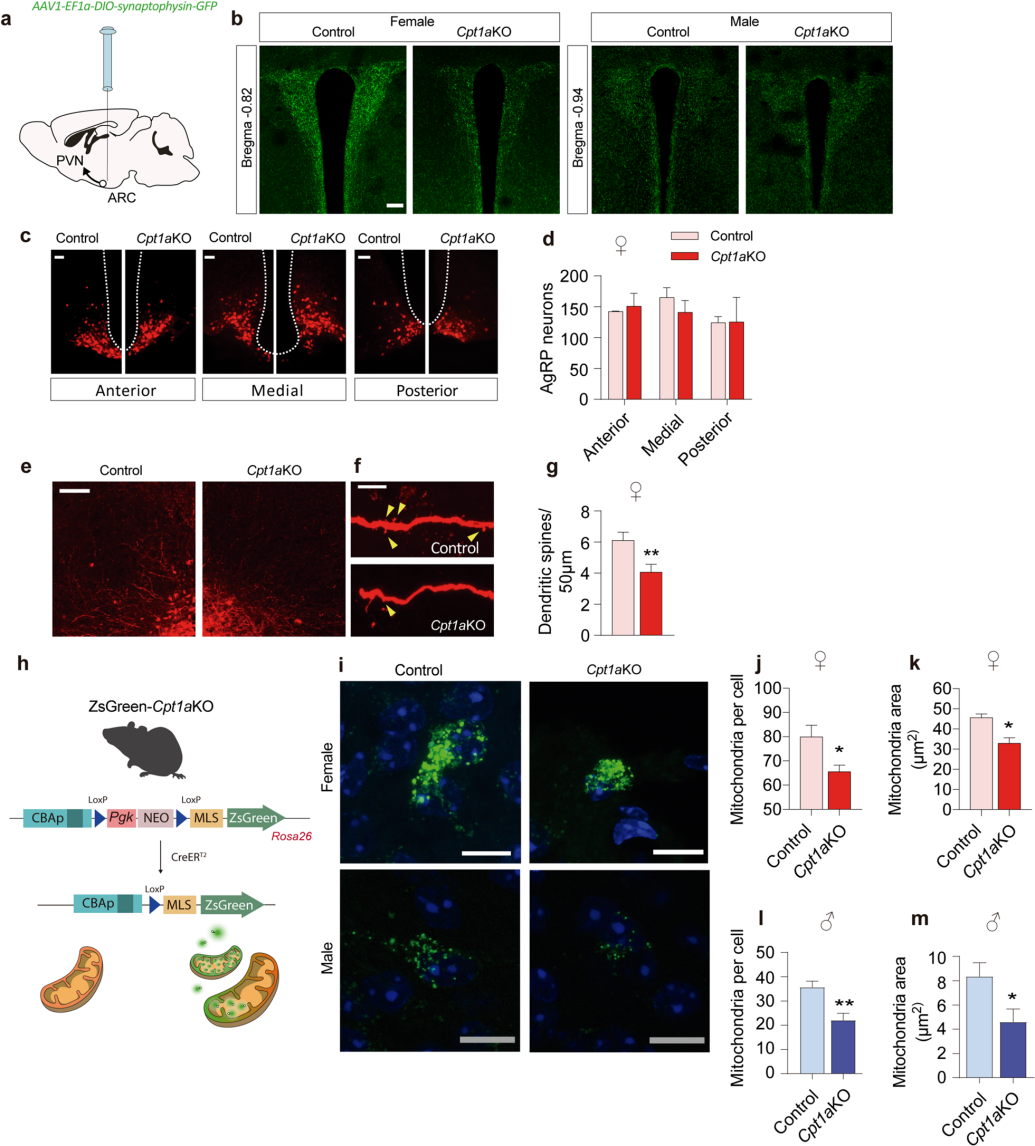
AgRP neurons lacking *Cpt1a* show reduced dendritic spines and projections to the PVN. **a** Scheme of *AAV1-EF1a-DIO-synaptophysin-GFP* administration into the ARC and the projection to the PVN. **b** Representative fluorescence microphotograph of AgRP-syn projection to the PVN in female and male mice. Scale bar, 10 μm. Syn-GFP intensity level was quantified with ImageJ using 6 slices (*n* = 3–4). **c** Representative images of AgRP neurons along the anterior–posterior axis of the ARC. **d** Quantification of AgRP neurons in the anterior, medial and posterior section of the ARC in female mice (**d**, *n* = 3–4). Scale bar, 25 μm. **e** Representative microphotograph of the anterior ARC section in female mice. Scale bar, 100 μm. **f**, **g** Representative fluorescence microphotograph of the dendrites of AgRP neurons. Scale bar, 100 μm. Quantification of the number of dendritic spines per 50 μm of dendrites in 20/25 axons of AgRP neurons in female mice (**g**, *n* = 3). **h** Scheme of mitochondrial labeling in the ZsGreen *Cpt1a*KO mice. **i** Representative fluorescence microphotograph of mitochondria from ZsGreen mice. Scale bar, 10 μm. **j**, **l** Quantification of the number of mitochondria per cell. **k**, **m** The average mitochondrial area in AgRP neurons (*n* = 3, 20–28 neurons per genotype). Data are expressed as the mean ± SEM. In **d**, **g**, **j**–**o**, *p < 0.05, **p < 0.01, ****p < 0.001 using Student’s *t*-test

Next, we studied the projections from AgRP neurons to the PVN in female *Cpt1a*KO mice under fasting conditions. To study this, we injected AAVs that conditionally expressed synaptophysin-GFP (*AAV1-EF1a-DIO-synaptophysin-GFP*) under Cre recombinase activation into the ARC. GFP fluorescence was analyzed in the presynaptic area of AgRP neurons in the PVN sections (Fig. 6a and b). We observed a reduction in the GFP fluorescence in the PVN section from *Cpt1a*KO female mice compared to their control littermates. This reduction was less observed in *Cpt1a*KO male mice. Altogether, this suggested that *Cpt1a* deletion in AgRP neurons reduced the projections from AgRP neurons to the PVN in female mice.

To explore if these reduced synapses were a result of reduced AgRP neuron viability, we analyzed the number of AgRP neurons along the anterior–posterior axis of the ARC. To accomplish this, we injected AAVs that conditionally expressed mCherry (*AAV9-EF1a-DIO-mCherry*) in the presence of Cre recombinase into the ARC of control mice or mice lacking *Cpt1a* in their AgRP neurons. Female mice were analyzed 1 month after tamoxifen induction. Importantly, no differences in the number of AgRP neurons were observed in the sections selected from the anterior, medial and posterior ARC (Fig. 6c and d). When we analyzed the morphology of AgRP neurons by confocal microscopy, we observed an alteration in the dendritic morphology of AgRP neurons lacking *Cpt1a* (Fig. 6e and g), which showed a reduced number of dendritic spines per 50 µm of dendrons compared to the AgRP neurons from their control littermates.

### Cpt1aKO mice showed altered mitochondria density and gene expression in AgRP neurons

To further investigate the mechanisms reducing the dendritic spines and presynaptic terminals, we first focused our attention on mitochondria. It has been reported that mitochondria play a key role in the number and size of dendritic spines [54]. We analyzed the effect of *Cpt1a* deletion in AgRP neurons on mitochondrial morphology in in vivo conditions. We first generated ZsGreen mice. *Cpt1a*KO mice were then crossed with ZsGreen mice that expressed the green fluorescent protein ZSGreen in the mitochondria of AgRP neurons after tamoxifen induction. Brain sections from *Cpt1a*KO-ZsGreen mice and their control littermates were obtained and analyzed by confocal microscopy. We observed an increased number and size of the mitochondria per cell of female with respect to the mitochondria per cell of males. Both *Cpt1a*KO-ZsGreen mice sexes showed a reduced number of mitochondria per cell as well as a reduced mitochondrial area (Fig. 6h–m). Although the lack of CPT1A reduced the number of mitochondria, there seemed to be enough mitochondria to support neuronal survival.

**Fig. 7:**
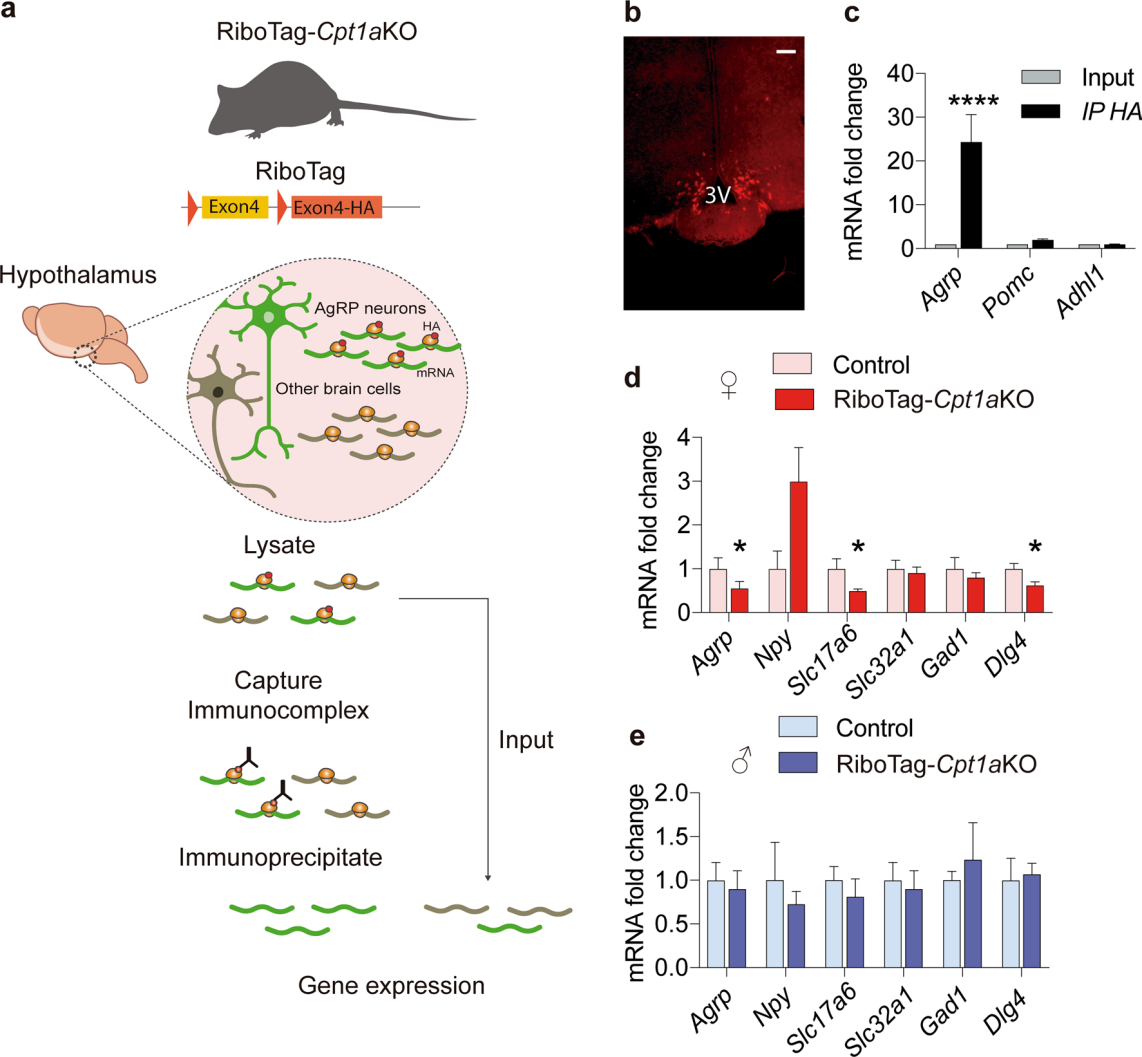
Validation of the RiboTag*Cpt1a*KO mouse model and gene expression analysis. **a** Scheme of the experimental strategy to isolate AgRP neuron-specific RNAs from *Cpt1a*KO–RiboTag mice. **b** Representative images of AgRP neurons tagged with the anti-HA antibody. Scale bar, 100 µm. **c** Analysis by qRT-PCR of mRNA levels of *Agrp, Pomc* and *Adh1* was performed in the input and immunoprecipitated HA samples. **d**, **e** Analysis by qRT-PCR of the mRNA levels of *Agrp, Npy, Slc17a6, Slc32a1, Gad1, Dlg4* in female mice (*n* = 4–6) and male mice (*n* = 5–6). The reference gene was *Gapdh* for all the samples analyzed by qRT-PCR*.* Data are expressed as the mean ± SEM. *p < 0.05, ****p < 0.0001, using Student’s *t*-test

To determine if this reduced pool of mitochondria and synapses affected the synthesis of the NPY and AgRP neuropeptides and neurotransmitters (GABA and glutamate) by AgRP neurons, we performed an in vivo transcriptomic analysis using the RiboTag technique [42]. We crossed *Cpt1a*KO mice with RiboTag homozygous mice. This allowed us to isolate AgRP neuron-specific ribosome-associated mRNAs from the ARC of RiboTag-*Cpt1a*KO mice by immunoprecipitating the actively translating polyribosomes tagged with the HA epitope (Fig. 7a). To confirm the correct labeling of the AgRP ribosomes, brain sections from RiboTag *Cpt1a*KO mice were obtained and we assessed the HA epitope by immunofluorescence (Fig. 7b). To determine the specificity of the immunoprecipitation for AgRP ribosomes, we evaluated the transcript levels of *Agrp, Pomc* and *Adhl1* as markers of AgRP neurons, POMC neurons and astrocytes, respectively, in the input and immunoprecipitate by qRT-PCR. We observed an increase in *Agrp* mRNA levels only in the immunoprecipitate, indicating the specific isolation of AgRP ribosomes (Fig. 7c). Gene expression analysis of the immunoprecipitate from RiboTag *Cpt1a*KO mice and their control littermates showed a reduction of mRNA levels of three genes, *Agrp,Slc17a6 and Dgl4* in the RiboTag *Cpt1a*KO female mice (Fig. 7d)*. Slc17a6* codifies for the protein of vesicular glutamate transporter (VGLU2) in presynaptic terminal and, while *Dgl4* codifies for a postsynaptic scaffolding protein (PSD-95) that plays a critical role in synaptogenesis and synaptic plasticity by providing a platform for the postsynaptic clustering of crucial synaptic proteins. No changes were observed in the gene expression of *Slc32a1* that codifies for vesicular transporter of GABA (VGAT1) or *Gad1* (glutamate descarboxylase), an enzyme involved in the synthesis of GABA (Fig. 7d). These changes were not observed in the males (Fig. 7e). All these results indicated that FA metabolism and CPT1A are involved in the neuronal processes related to neurotransmission and neuropeptide expression in AgRP neurons in fasting conditions and play different roles depending on sex.

## Discussion

In this study, we showed that CPT1A, an enzyme that regulates FAO, is relevant for orexigenic AgRP neuronal function, energy homeostasis, and fluid balance in a sex-dependent way (Table 1). Conditional *Cpt1a* deletion in AgRP neurons in adult mice led to a clear reduction in body weight gain in *Cpt1a*KO male mice, which was less pronounced in the female mice compared to their control littermates. These sex-dependent differences were clearer in the feeding behavior, since *Cpt1a*KO female mice did not show any change in food intake under ad libitum conditions. Sex-based differences in feeding behavior in mammals have been attributed exclusively to the effects of gonadal hormones, especially estrogens and androgens, which regulate food intake and energy metabolism by acting on the brain and diverse peripheral tissues [55–57]. It has been shown that in gonadal-intact mice, total food intake is higher in male than in female mice during dark phases, with female mice eating more than the males during light phases. Interestingly, these differences were blunted in gonadectomized animals, since no differences were observed between the sexes [58], thus suggesting a complex interaction between the gonadal hormones in daily feeding rhythms and revealing sex differences depending on gonadal hormones. In our model, *Cpt1a* deletion in AgRP neurons affected food intake during the dark phase in male *Cpt1a*KO mice, which dropped by half.

**Table 1:** Summary of metabolic phenotypes between males and females and sexual dimorphism

Parameter	Control Female	*Cpt1a*KO Female	Control Male	*Cpt1a*KO Male	Sexual dimorphism
Body weight gain	–	↓	–	↓	
Cumulative food intake	–	–	–	↓	*
Food intake induced by fast	–	↓	–	↓	
Food intake induced by ghrelin	–	↓	↓	↓↓	*
Energy expenditure	–	↑	–	–	*
BAT temperature	–	↑	↑	–	*
BAT tissue	–	↓	–	↓	
Lipid droplet area	–	↓	–	↓	
Thermogenic gene expression in BAT	–	↑	–	↑	
UCP1 protein	–	↑	–	↑	
gWAT tissue	–	↓	–	↓	
adipocyte size of gWAT	–	↓	–	↓	
adipocyte size of iWAT	–	↓	–	↓	
TG and NEFAs	–	↓	–	↓	
Leptin	–	–	–	–	
UCP1 gene expression in iWAT	–	↑↑	–	↑	
Urine output	–	↑	–	↑	
Urine osmolality under water ad libitum	–	nd	–	↓	
Water intake	–	↑	–	↑	
Plasma osmolality	–	–	–	–	
Vasopressin	–	↓	↑	↓	*
Renin	–	↑	–	↑	
Angiotensin II	–	↑	–	↑	
Aldosterone	–	↑	–	↑	
Systolic blood pressure	–	↑	–	↑	
GTT (AUC)	–	–	–	↓	*
ITT	–	–	–	–	
Fasted insulin	–	–	–	–	
Urine glucose	–	–	–	–	
Liver weight	–	–	–	–	
*Cpt1a* liver expression	–	↑	–	↑	
*Ucp2, Pepck* and *G6pc*	–	–	–	↑	*
Mitochondrial content	–	↓	–	↓	
*Agrp, Slc17ab* and *Dlg4* gene expresion in AgRP neurons	–	↓	–	–	*
17β-Estradiol	–	–			
Testosterone			–	↓	

*Indicates sexual dimorphism considering control females as reference. nd, not detected

It has been reported that the fasting-mediated activation of AgRP neurons promotes food-seeking behavior and energy conservation [59]. In *Cpt1a*KO mice, we observed an important reduction in food intake in challenging conditions, such as during fasting-induced refeeding and ghrelin administration, in both sexes. These results suggested that neurons lacking CPT1A suffer metabolic inflexibility. Thus, AgRP neurons cannot cope with the transition between fasting and refeeding. These findings agree with those of two previous studies [60, 61]. In agreement with these findings, our results suggested that CPT1A and FA metabolism are imperative in the feeding behavior response after the activation of AgRP neurons during fasting. In addition, it has been observed that ghrelin, a hormone that stimulates appetite, binds to its receptor [62–64] and promotes feeding through the activation of the AMPK–CPT1A–UCP2 axis in hypothalamic neurons [65–67]. Although the effects of ghrelin on AgRP neurons are well established, it is not clear which molecular mechanisms and pathways are involved in AgRP neuron activation. Our finding reinforces the importance of CPT1A downstream of the ghrelin receptor in AgRP neurons for the induction of food-seeking behavior, since both female and male mice lacking CPT1A in their AgRP neurons reduced food intake after ghrelin administration.

Although the reduction in body weight gain triggered by the *Cpt1a* deletion in AgRP neurons was independent of sex, our findings provide evidence that males and females are hardwired differently in the regulation of their energy balance. At the physiological level, *Cpt1a*KO male mice reduced their body weight gain through dietary restriction more efficiently than the female mice, while *Cpt1a*KO female mice increased EE that exceeded the energy ingested. A change in food consumption affects EE primarily through its effect on diet-induced EE [68]. Thus, a decrease in food intake decreases EE and vice versa. Accordingly, the drop in the food intake observed in *Cpt1a*KO male mice probably blunted the increase of the EE observed in *Cpt1a*KO female mice. This increased EE in *Cpt1a*KO female mice correlated with an exacerbated thermogenesis in their BAT, an enhanced browning of their subcutaneous adipose tissue (iWAT) and increased lipolytic activity in their adipose tissues, resulting in an important reduction in their fat mass. The connection between AgRP neurons and BAT has been well established, anatomically and functionally [69–71]. The results from our model are suggestive that *Cpt1a* deletion in AgRP neurons triggers an increase in the sympathetic outflow to BAT. *Cpt1a*KO mice presented enhanced BAT activity, showing an increased temperature of the suprascapular area mainly in the females. This was confirmed by measuring UCP1 protein levels in BAT and the expression of the genes encoding lipolytic enzymes. In addition, an enhanced browning of iWAT and an important reduction in the fat pads, mainly in the females, were consistent with the physiological role of AgRP neurons in the regulation of peripheral nutrient utilization [7, 72–74].

The liver is also an essential target of AgRP neurons in the maintenance of energy homeostasis [61, 75]. This agrees with our findings from the gene expression analysis of the livers of *Cpt1a*KO mice. Both male and female *Cpt1a*KO mice showed a significant increase in *Cpt1a* gene expression in the liver, suggestive of increased β-oxidation of FA. We also observed a significant increase in the expression of *Pepck* and *G6pase* in the male but not female mice under fasting conditions, suggesting a new sex-dependent difference in coping with challenging fasting conditions.

Most studies on AgRP function have focused on the molecular mechanisms underlying food intake, energy metabolism and body weight changes. However, little is known about the implication of AgRP in water consumption. The polydipsia and polyuria observed in the *Cpt1a*KO mice were consistent with the reduced blood levels of VP/ADH in *Cpt1a*KO mice compared to their control littermates. Considering that the PVN is an important nucleus for VP/ADH production and since AgRP neurons project to the PVN, the observed VP/ADH reduction could be due to an impaired ability of AgRP neurons to activate the PVN. We also investigated the RAAS, which is a critical regulator of fluid balance and blood pressure. Typically, the RAAS is activated when there is a drop in the blood volume to increase water and electrolyte reabsorption in the kidneys. We measured the plasma levels of AgII to determine whether the polyuria activated the RAAS. The increased blood levels of AgII observed in *Cpt1a*KO mice indicated that the normal renal parenchyma was able to detect the drop in water and electrolyte reabsorption, triggering RAAS activation. Thus, this cascade emerges as a compensatory mechanism for water loss. Our finding was supported by those of studies evaluating the effect of AngII on diuresis, since the intravenous infusion of angiotensin inhibited polyuria [76]. It is also plausible to speculate a direct activation of renin release from the juxtaglomerular cells of the kidneys. It is well stablished that the SNS triggers renin release for the generation of angiotensin I, which is then converted to Ang II [77]. More recently, direct multifiber recording of sympathetic nerve activity subserving the kidney was performed followed by a chemogenetic activation of AgRP neurons. The authors showed a rapid decline of sympathetic nerve activity upon AgRP activation [78]. Based on the experiments reported here, the lack of CPT1A in AgRP neurons is expected to upregulate sympathetic nerve activity to the kidney. This could also account for the activation of RAAS in our model. AgII raises blood pressure through different mechanisms, the most important ones being vasoconstriction, sympathetic nervous stimulation, increased aldosterone biosynthesis and renal activities. Here, we observed that both systolic blood pressure and aldosterone levels were increased in *Cpt1a*KO mice, strongly reinforcing RAAS activation. AgII has also been reported to exert effects on the brain. It can bind to the hypothalamus, stimulating thirst and the release of VP/ADH by the posterior pituitary. However, the reduced level of this hormone in *Cpt1a*KO mice indicated that AgII requires neuronal stimuli to maintain VP/ADH levels under physiological conditions. Altogether, our results suggested that the normal function of AgRP neurons is necessary to maintain fluid balance.

The drop in the activation of PVN neurons suggested reduced AgRP presynaptic innervation or neuronal viability. We explored both and did not find differences in the number of AgRP neurons in the ARC sections analyzed. However, the number of dendritic spines on AgRP neurons were reduced, as in the studies reporting a lack of NMDAR signaling components in spines [8, 60]. The analysis of AgRP presynaptic terminals in the PVN using labeled synaptophysin specifically expressed in AgRP neurons confirmed that female *Cpt1a*KO mice substantially reduced their presynaptic activity, which occurred to a lesser extent in the males. This suggested that the female mice were very susceptible to the lack of the CPT1A enzyme. One limitation of this study is to understand which specific neurons of PNV or other hypothalamic nucleus could be affected by the reduced AgRP presynaptic activity in both sexes. This could potentially explain the physiological sex differences observed not only in the maintenance of the fluid balance, but also in the control of food intake and body weight.

Mitochondria play important roles in neurons. In addition to energy production via the synthesis of ATP, this dynamic organelle is involved in cellular metabolism, innate and adaptative immune responses, and cell death (revised in [79]). Since CPT1A is a key enzyme in FAO, we speculated that the lack of CPT1A enzyme in AgRP neurons may produce metabolic shifts that could affect spine formation and synapses. Interestingly, we have found sex differences in the size and quantity of mitochondria per neuron in control mice. These findings highlight the possibility that females could be more protected to cope with stressful conditions [80, 81]. We also observed a reduced size and number of mitochondria per neuron in mice lacking CPT1A, mainly in females, which could reduce the energy production necessary for many neuronal processes. In line with this, in culture of embryonic primary cortical neurons we have observed that the lack of CPT1A reduces GABA release [40]. Another limitation of our study is the lack of analysis of the functionality that reduced mitochondria have on neuronal processes in adult mice of both sexes in vivo*.* In order to do this*,* the AgRP neuronal isolation and culture from adult mice would be needed, but currently primary hypothalamic cultures are only feasible in the embryonic stage. In a recent study in embryonic hypothalamic culture from mice with a AgRP-selective deletion of *Dnm1*, which codifies for a mediator of mitochondrial fission protein (DRP1), has been shown an attenuated mitochondrial respiration that could affect AgRP function [82]. Future studies assessing the mitochondrial functionality and subcellular distribution in both sexes are necessary to clarify the mitochondrial role in the AgRP neuronal function.

RiboTag analysis also showed important differences in gene expression between the females and males. In fasting conditions, female mice showed reduced transcript levels of key genes involved in neurotransmission and neuropeptide production, which was not observed in the males. The decrease in AgRP neuropeptide levels was consistent with the increased EE observed in *Cpt1a*KO female mice. Neurons in the PVN expressing thyroid-releasing hormone (TRH), oxytocin (OT), and corticotropin-releasing hormone (CRH) all express MC4R [83]. The binding of α-MSH secreted by POMC neurons to MC4R on these neurons has a positive effect on the hypothalamic–pituitary–thyroid (HPT) axis and the hypothalamic–corticotropic axis (HPA) [84]. Since AgRP acts as an inverse agonist of α-MSH in the PVN in fasting conditions [85–88] its reduced expression in *Cpt1a*KO female mice could stimulate both the HPT and HPA axes, resulting in a positive enhancement of thermogenesis and EE. However, other brain regions and neuronal mechanisms could be involved in the enhancement of thermogenic activity, EE and the control of body weight, since specific groups of AgRP neurons have been reported to project to the dorsal lateral part of the dorsal raphe nucleus [89] and the parabrachial nucleus [90]. We also observed reduced *Slc17a6* mRNA levels in *Cpt1a*KO female mice, which could partly explain the reduced activity of vasopressin neurons. Glutamate and GABA are the main neurotransmitters in the PVN and SON that are involved in the synaptic regulation of vasopressin neurons [91]. A decrease in glutamatergic inputs is partly consistent with the polydipsia and polyuria observed in our *Cpt1a*KO mouse model. Although there were no changes in VGAT gene expression in fasting conditions between control and *Cpt1a*KO mice, we cannot discard the effect of GABA in polydipsia in conditions other than fasting [90]. *Dgl4* mRNA levels were also decreased in female *Cpt1a*KO mice. Since PSD-95, the protein codified by *Dgl4* gene, is required for the stabilization of spines, the maturation of excitatory synapses and the synaptic function [92], we propose that this reduction on *Dgl4* mRNA levels could result in a partial reduction of the postsynaptic plasticity of AgRP neurons in fasting conditions. The decrease of both *Scl17a6* and *Dgl4* mRNA levels suggest that *Cpt1a*KO female mice reduce AgRP neuronal synaptic processes in the fasting conditions, which is mildly observed in *Cpt1a*KO male mice. This is consistent with the reduced number of mitochondria, presynaptic innervation to PVN observed in females in respect to the males. We propose that this reduction in the neuropeptide AgRP and proteins involved synaptic processes could reduce the signaling to the PVN enhancing HPA axis and sympathetic tone in fasting conditions.

Sexual hormones could be affected by AgRP levels. It has been shown that starvation-activated agouti-related peptide (AgRP) neurons can inhibit the reproductive neuroendocrine circuit, mainly in females [93]. No significant changes were observed in 17β-estradiol in *Cpt1a*KO female mice, in contrast, *Cpt1a*KO male mice showed a decrease of testosterone. Since levels of testosterone are mediated by the hypothalamic–pituitary–gonadal axis we cannot rule out that a drop in the activation of the PVN neurons from AgRP neurons could modulate the hypothalamic releases of gonadotropin-releasing hormone (GnRH) and further reducing the secretion of follicle-stimulating hormone (FSH) and luteinizing hormone (LH) by the anterior pituitary gland and consequently leading a reduction of testosterone production. All these results highlight the different role of AgRP neurons in males and females.

## Perspectives and significance

Our study provided insight into understanding how fatty acid metabolism and, in particular, CPT1A in AgRP neurons play role in the control of food intake, energy expenditure and body weight. We suggest that CPT1A should be considered as a new target against obesity. Additionally, we provide evidence that AgRP neurons are involved in the control of thirst and fluid balance, suggesting that AgRP neurons are relevant components of the neural circuits underlying thirst and fluid homeostasis. Further analysis of AgRP connections with other centers of the hypothalamus and the brain that are involved in the control of thirst could help to understand the circuitry and the mechanisms that underlie drinking behavior.

Our study demonstrates that there are sex-specific differences in the effect of CPT1A deletion, a key enzyme in fatty acid metabolism, on specific neurons, emphasizing the need to analyze both females and males when describing the mouse phenotypes. Future studies will be needed to specifically address the relevance of fatty metabolism in mitochondrial function and consequently in neuronal processes.

## Supplementary Information

**Additional file 1: Figure S1.** Validation of Cre-mediated recombination in AgRP neurons. **a** Scheme of the time-course of the experiment. **b** Bilateral injection of 400 nl of AAV9-EF1a-DIO-mCherry at a dose of 1.23 × 10^13^ gc/ml into the ARC of AgRP-Cre-ERT2 mice. **c** Representative histological slice of Cre-dependent mCherry expression in the ARC. Scale bar, 500 μm (top image) and 200 μm (bottom image). **d** Schematic of the Cre-mediated recombination product showing the floxed band (1030 bp) containing the LoxP sequences surrounding exon 4 of the *Cpt1a* gene. After Cre recombination, the product resulted in a 219-bp DNA fragment. **e** Scheme of the restriction sites of the restriction enzymes *PstI* and *AatII* that were used to separate *Cpt1a* amplicons from unrecombined genomic DNA. **f** Representative PCR analysis of genomic DNA from the liver, gonadal white adipose tissue (gWAT), brown adipose tissue (BAT), adrenal gland (AG), cortex, hippocampus (Hyp) and arcuate nucleus (ARC) treated with *Pst*I and *Aat*II enzymes in Cpt1a*KO* mice and control mice **(g)** FASTA analysis of the sequenced 219 bp DNA fragment extracted from the gel.

**Additional file 2: Figure S2.** Analysis of the adrenal gland after *Cpt1a* deletion in AgRP neurons. **a** Representative H&E staining of female (left panel) and male adrenal gland (right panel). Scale bar, 500 μm (magnification 4×) and 100 μm (magnification 20×). **b** and **c** Weight of the left and right adrenal glands in female (b, *n* = 5–5) and male mice (c, *n* = 5–5). (**d** and **e**) Analysis by qRT-PCR of the mRNA levels of *Th* in the adrenal gland of female (D, *n* = 5–9) and male mice (E, *n* = 9–7).

**Additional file 3: Figure S3.** Effect of *Cpt1a* deletion on EE. **a** Representative image of Cpt1aKO female (left image) and male mice (right image) compared with their control littermates 3 months after tamoxifen induction. **b** and **c** 24 h of food consumption in female (b, n = 8–10) and male mice (c, *n* = 6–9). **d** and **e** EE in light and dark cycles and during fasting and refeeding in female (d, *n* = 6–6) and male mice (e, *n* = 6–6). **f** EE in light and dark cycles comparing control littermates and *Cpt1a*KO animals (f, *n* = 6–6). **g** and **h** Respiratory quotient (RQ) registered by the TSE system in female (f, *n* = 6–6) and male mice (g, *n* = 6–6). **h** and **i** Locomotor activity (LA) registered by the TSE system in female (h, *n* = 6–6) and male mice (i, *n* = 6–6). Data are expressed as the mean ± SEM. In b–i, * *p* < 0.05, using Student’s *t*-test.

**Additional file 4: Figure S4.** Deletion of *Cpt1a* in AgRP neurons reduces the fat mass in male and female mice. **a** and **b** Gonadal white adipose tissue (gWAT) weight in female (a, *n* = 6) and male mice (b, *n* = 6). (**c** and **d**) (**c** and **d**) Plasma level of leptin in female (c, *n* = 4–5) and male mice (d, *n* = 4–7). **e** and **f** Analysis by qRT-PCR of *Pnpla2, Vegfa, Il6, Leptin, Mmp2, Adiponectin* and *Resistin* mRNA levels in the gWAT of female (e, *n* = 8–6) and male mice (f, *n* = 9–6). Data are expressed as the mean ± SEM. In a–d, * *p* < 0.05, using Student’s *t*-test.

**Additional file 5: Figure S5**. Deletion of *Cpt1a* in AgRP neurons upregulates *Cpt1a* expression in the liver. **a** Representative hematoxylin and eosin (H&E) staining of the livers of male and female mice. Scale bar, 50 µm (magnification 20×). **b** and **d** Liver weight of female (b, *n* = 6–8) and male mice (d, *n* = 6–8). (**c and e**) Analysis by qRT-PCR of *Cpt1a, Cd36, Ucp2, Hmgcs2, Pepck* and *G6pc* mRNA levels in female (c, n = 8) and male mice (e, *n* = 9–8). **f** Representative H&E staining of the pancreas and testes from male mice (upper panel) and the pancreas and ovaries from female mice (lower panel). **g** Plasma level of 17β-estradiol in female mice (*n* = 8–8) and **h** testosterone level in male mice (*n* = 9–10). Scale bar, 50 µm (magnification 20×). Data are expressed as the mean ± SEM. In b–e, * *p* < 0.05, using Student’s *t*-test.

**Additional file 6: Figure S6.** *Cpt1a* deletion in AgRP neurons does not induce a diabetic state. **a** and **f** Glucose tolerance test (GTT) in female (a, *n* = 10–8) and male mice (f, *n* = 10–8). **b** and **g** area under the curve (AUC) quantification in female (b, *n* = 10–8) and male mice (g, *n* = 10–8). **c** and **h** Insulin tolerance test (ITT) in female (c, *n* = 7–8) and male mice (h, *n* = 10–9). **d** and **i** Fasting insulin levels in female (d, *n* = 8–6) and male mice (i, *n* = 7–4). **e** and **j** Urinary glucose levels in female (e, *n* = 5–7) and male mice (j, *n* = 9–6). **k** Representative hematoxylin and eosin (H&E) staining of the cortex (left panel) and medulla (right panel) of the kidneys from female and male mice. Scale bar, 50 µm (magnification 20×). Data are expressed as the mean ± SEM. In a, f, c, h, * *p* < 0.05, using two-way repeated-measures ANOVA followed by Šidák’s post hoc test. In b, g, d–j, *** *p* < 0.001, using Student’s *t*-test.

**Additional file 7.** **Table S1:** Forward and reverse primers used in the PCR analysis. **Table S2:** Antibodies used in the study.

## Data Availability

The datasets generated during and/or analyzed during the current study are not publicly available due to the Universitat of Barcelona do not have a repository for research data but are available from the corresponding author on reasonable request.

## Abbreviations

AAVs: Adeno-associated viruses
ACC: Acetyl-CoA carboxylase
AgRP: Agouti-related peptide
AMPK: AMP-activated protein kinase
AgII: Angiotensin II
ARC: Arcuate nucleus
BAT: Brown adipose tissue
Cidea: Cell death inducing DFFA like effector A
CPT1A: Carnitine palmitoyltransferase 1A
Dlg4: Discs large MAGUK scaffold protein 4
EE: Energy expenditure
FA: Fatty acid
Dnm1: Dynamin-1
DRP1: Dynamin-related protein 1
FAO: Fatty acid oxidation
FAS: Fatty acid synthase
GABA: Gamma-aminobutyric acid
GAD: Glutamate decarboxylase
Gapdh: Glyceraldehyde-3-phosphate dehydrogenase
G6pc: Glucose 6-phosphatase
GTT: Glucose tolerance test
Hmgcs2: 3-Hydroxy-3-methylglutaryl-CoA synthase 2
iGluR: Ionotropic glutamate receptor
gWAT: Gonadal white adipose tissue
LA: Locomotor activity
LCFA: Long-chain fatty acid
MCD: Malonyl-CoA decarboxylase
MTS: Mitochondrial targeting signal
Hprt: Hypoxanthine–guanine phosphoribosyl-transferase
ITT: Insulin tolerance test
Lipe: Lipase E
iWAT: Inguinal white adipose tissue
Mmp2: Matrix metallopeptidase 2
MCD: Malonyl-CoA decarboxylase
NEFA: Non-esterified fatty acid
NPY: Neuropeptide Y
PFA: Paraformaldehyde
PBS: Phosphate buffered saline
PCR: Polymerase chain reaction
PEPCK: Phosphoenolpyruvate carboxykinase
Pnpla2: Patatin-like phospholipase domain-containing 2
POMC: Proopiomelanocortin
PVN: Paraventricular nucleus
RAAS: Renin–angiotensin–aldosterone system
RPL22HA: Ribosomal protein L22-hemagglutinin
RQ: Respiratory quotient
Slc2a: Solute carrier family 2 member 4
Slc32a1: Solute carrier family 32 member 1
Slc17a6: Solute carrier family 17 member 6
SNS: Sympathetic nervous system
SON: Supraoptic nucleus
TGs: Triglycerides
Th: Tyrosine hydroxylase
UCP: Uncoupling protein
VP/ADH: Vasopressin
WT: Wild type
