# Supplementary figures and images for: CPT1A in AgRP neurons is required for sex-dependent regulation of feeding and thirst
Source: Biol Sex Differ. 2023 Mar 25;14:14. doi: 10.1186/s13293-023-00498-8 (PMC10040140; doi:10.1186/s13293-023-00498-8)

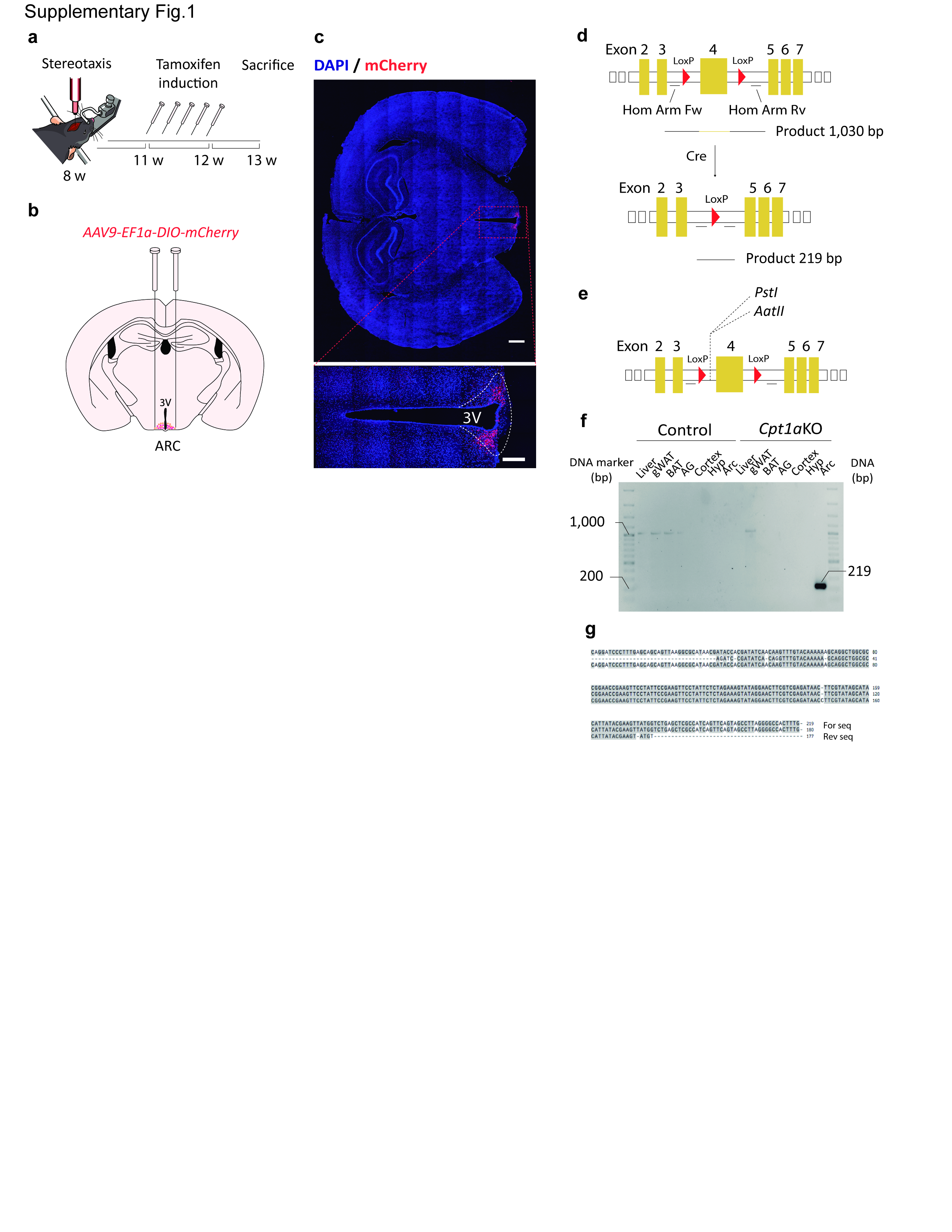

Supplement: Supplementary file 1 — Additional file 1: Figure S1. Validation of Cre-mediated recombination in AgRP neurons. a Scheme of the time-course of the experiment. b Bilateral injection of 400 nl of AAV9-EF1a-DIO-mCherry at a dose of 1.23 × 10^13^ gc/ml into the ARC of AgRP-Cre-ERT2 mice. c Representative histological slice of Cre-dependent mCherry expression in the ARC. Scale bar, 500 μm (top image) and 200 μm (bottom image). d Schematic of the Cre-mediated recombination product showing the floxed band (1030 bp) containing the LoxP sequences surrounding exon 4 of the Cpt1a gene. After Cre recombination, the product resulted in a 219-bp DNA fragment. e Scheme of the restriction sites of the restriction enzymes PstI and AatII that were used to separate Cpt1a amplicons from unrecombined genomic DNA. f Representative PCR analysis of genomic DNA from the liver, gonadal white adipose tissue (gWAT), brown adipose tissue (BAT), adrenal gland (AG), cortex, hippocampus (Hyp) and arcuate nucleus (ARC) treated with PstI and AatII enzymes in Cpt1aKO mice and control mice (g) FASTA analysis of the sequenced 219 bp DNA fragment extracted from the gel. [file 13293_2023_498_MOESM1_ESM.tif]

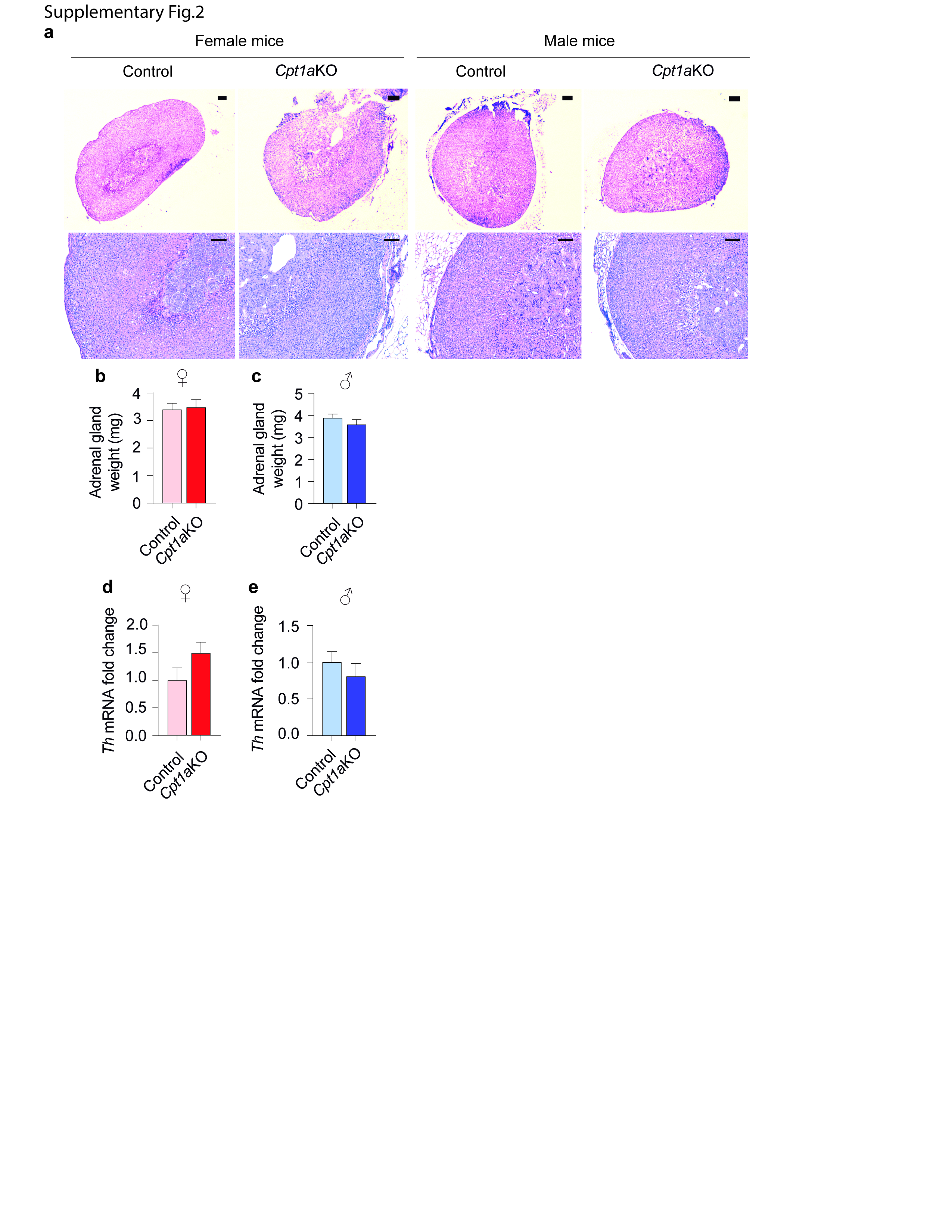

Supplement: Supplementary file 2 — Additional file 2: Figure S2. Analysis of the adrenal gland after Cpt1a deletion in AgRP neurons. a Representative H&E staining of female (left panel) and male adrenal gland (right panel). Scale bar, 500 μm (magnification 4×) and 100 μm (magnification 20×). b and c Weight of the left and right adrenal glands in female (b, n = 5–5) and male mice (c, n = 5–5). (d and e) Analysis by qRT-PCR of the mRNA levels of Th in the adrenal gland of female (D, n = 5–9) and male mice (E, n = 9–7). [file 13293_2023_498_MOESM2_ESM.tif]

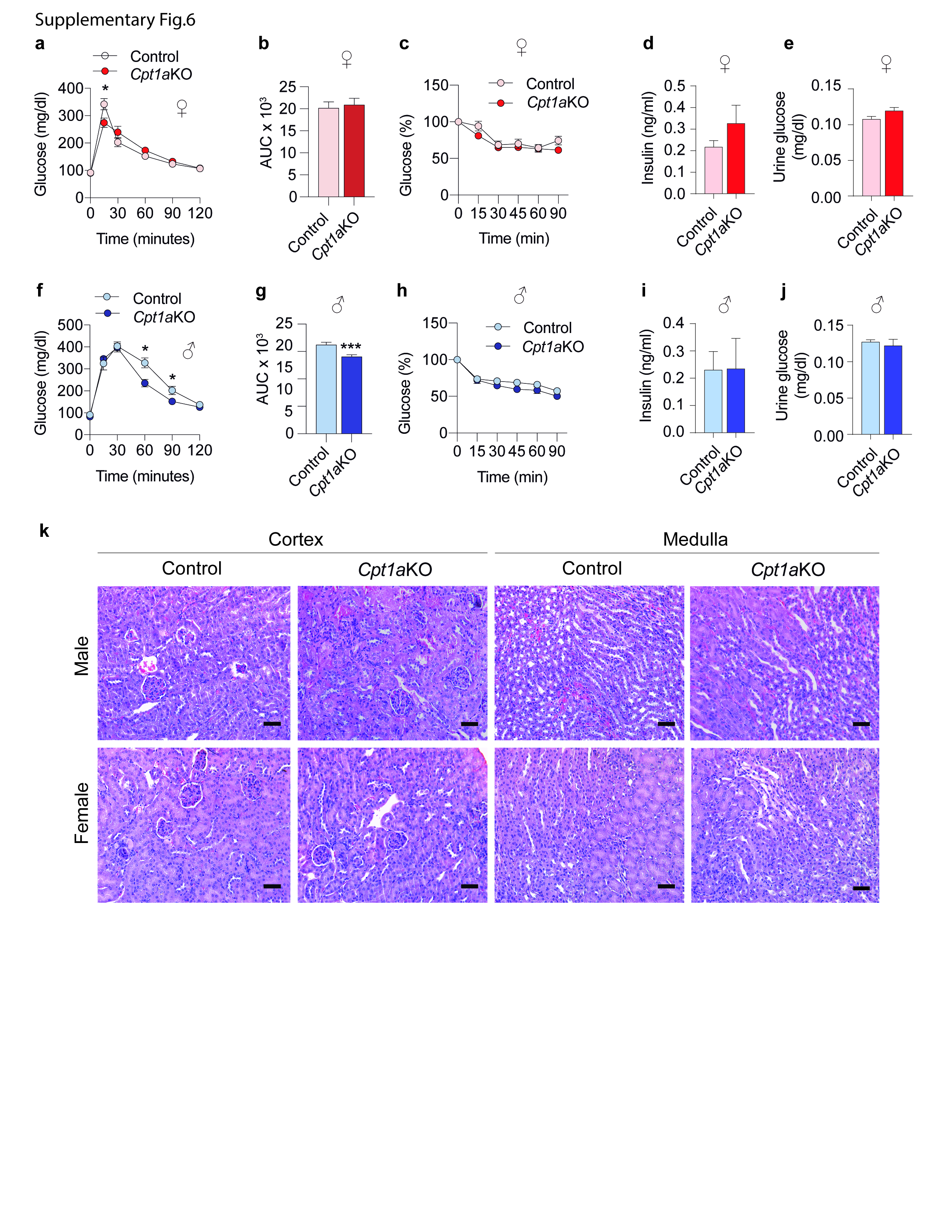

Supplement: Supplementary file 6 — Additional file 6: Figure S6. Cpt1a deletion in AgRP neurons does not induce a diabetic state. a and f Glucose tolerance test (GTT) in female (a, n = 10–8) and male mice (f, n = 10–8). b and g area under the curve (AUC) quantification in female (b, n = 10–8) and male mice (g, n = 10–8). c and h Insulin tolerance test (ITT) in female (c, n = 7–8) and male mice (h, n = 10–9). d and i Fasting insulin levels in female (d, n = 8–6) and male mice (i, n = 7–4). e and j Urinary glucose levels in female (e, n = 5–7) and male mice (j, n = 9–6). k Representative hematoxylin and eosin (H&E) staining of the cortex (left panel) and medulla (right panel) of the kidneys from female and male mice. Scale bar, 50 µm (magnification 20×). Data are expressed as the mean ± SEM. In a, f, c, h, * p < 0.05, using two-way repeated-measures ANOVA followed by Šidák’s post hoc test. In b, g, d–j, *** p < 0.001, using Student’s t-test. [file 13293_2023_498_MOESM6_ESM.tif]
