# Supplementary material for: CPT1A in AgRP neurons is required for sex-dependent regulation of feeding and thirst
Source: Biol Sex Differ. 2023 Mar 25;14:14. doi: 10.1186/s13293-023-00498-8 (PMC10040140; doi:10.1186/s13293-023-00498-8)
Supplement: Supplementary file 7 — Additional file 7. Table S1: Forward and reverse primers used in the PCR analysis. Table S2: Antibodies used in the study. [file 13293_2023_498_MOESM7_ESM.docx]

Table S1

| **GENE** | **SOURCE** | **SEQUENCE** |
| --- | --- | --- |
| *Cpt1a* forward | Sigma-Aldrich | 5’-GCTTATCGTGGTGGTGGGTGT-3’ |
| *Cpt1a* reverse | Sigma-Aldrich | 5’-GTTGACAGCAAAATCCTGGGC-3’ |
| *Th* forward | Sigma-Aldrich | 5’-TGTTGGCTGACCGCACAT-3’ |
| *Th* reverse | Sigma-Aldrich | 5’-GCCCCCAGAGATGCAAGT-3’ |
| *Leptin* forward | Sigma-Aldrich | 5’-CAGGATCAATGACATTTCACACA-3’ |
| *Leptin* reverse | Sigma-Aldrich | 5’-CAGGATCAATGACATTTCACACA-3’ |
| *Il6* forward | Sigma-Aldrich | 5’-CTGCAAGAGACTTCCATCCAGT-3’ |
| *Il6* reverse | Sigma-Aldrich | 5’-GAAGTAGGGAAGGCCGTGG-3’ |
| *Pnpl2* forward | Sigma-Aldrich | 5’-TGTAGGTGGCGCAAGACA-3’ |
| *Pnpl2* reverse | Sigma-Aldrich | 5’-TGTAGGTGGCGCAAGACA-3’ |
| *Lipe* reverse | Sigma-Aldrich | 5’-CGCTCTCCAGTTGAACCAAG-3’ |
| *Lipe* reverse | Sigma-Aldrich | 5’-CGCTCTCCAGTTGAACCAAG-3’ |
| *Cpt1b* forward | Sigma-Aldrich | 5’-TGCCTTTACATCGTCTCCAA-3’ |
| *Cpt1b* reverse | Sigma-Aldrich | 5’-TGCCTTTACATCGTCTCCAA-3’ |
| *Ucp1* forward | Sigma-Aldrich | 5’-GGCCTCTACGACTCAGTCCA-3’ |
| *Ucp1* reverse | Sigma-Aldrich | 5’-GGCCTCTACGACTCAGTCCA-3’ |
| *Cidea* forward | Sigma-Aldrich | 5’-GCCTGCAGGAACTTATCAGC-3’ |
| *Cidea* reverse | Sigma-Aldrich | 5’-AGAACTCCTCTGTGTCCACCA-3’ |
| *Mmp2* forward | Sigma-Aldrich | 5’-TAACCTGGATGCCGTCGT-3’ |
| *Mmp2* reverse | Sigma-Aldrich | 5’-TAACCTGGATGCCGTCGT-3’ |
| *Vegfa* forward | Sigma-Aldrich | 5’-GGCCTCTACGACTCAGTCCA-3’ |
| *Vegfa* reverse | Sigma-Aldrich | 5’-GGCCTCTACGACTCAGTCCA-3’ |
| *Hprt* forward | Sigma-Aldrich | 5’-TCCTCCTCAGACCGCTTTT-3’ |
| *Hprt* reverse | Sigma-Aldrich | 5’-TCCTCCTCAGACCGCTTTT-3’ |
| *18s* forwars | Sigma-Aldrich | 5’-TGGTTGATCCTGCCAGTAG-3’ |
| *18s* reverse | Sigma-Aldrich | 5’-CGACCAAAGGAACCATAACT-3’ |
| *Tbp* forward | Sigma-Aldrich | 5’-ACCCTTCACCAATGACTCCTATG-3’ |
| *Tbp* reverse | Sigma-Aldrich | 5’-TGACTGCAGCAAATCGCTTGG-3’ |
| *β-actin* forward | Sigma-Aldrich | 5’-AGGTGACAGCATTGCTTCTG-3’ |
| *β-actin* reverse | Sigma-Aldrich | 5’-GCTGCCTCAACACCTCAAC-3’ |
| *Rpl32* forward | Sigma-Aldrich | 5’-GCTGCCATCTGTTTTACGG-3’ |
| *Rpl32* reverse | Sigma-Aldrich | 5’-TGACTGGTGCCTGATGAACT-3’ |
| *Ucp2* forward | Sigma-Aldrich | 5’-CCGGGGCCTCTGGAAAG-3’ |
| *Ucp2* reverse | Sigma-Aldrich | 5’-CCCAAGCGGAGAAAGGA-3’ |
| *Hmgcs2* forward | Sigma-Aldrich | 5’-GTTGGAGTGTCTCTTACGGTTCTG-3’ |
| *Hmgcs2* reverse | Sigma-Aldrich | 5’-AGTTCTCGAGTCAAGCCTTGATTTA-3’ |
| *Pepck* forward | Sigma-Aldrich | 5’-GTCAACACCGACCTCCCTTA-3’ |
| *Pepck* reverse | Sigma-Aldrich | 5’-CCCTAGCCTGTTCTCTGTGC-3’ |
| *G6pc* forward | Sigma-Aldrich | 5’-AGGAAGGATGGAGGAAGGAA-3’ |
| *G6pc* reverse | Sigma-Aldrich | 5’-TGGAACCAGATGGGAAAGAG-3’ |
| *Slc2a4* forward | Sigma-Aldrich | 5’-GATGACCGTGGCTCTGCT-3’ |
| *Slc2a4* reverse | Sigma-Aldrich | 5’-GCTCTGCCACAATGAACCA-3’ |
| *Cd36* forward | Sigma-Aldrich | 5’-TTGTACCTATACTGTGGCTAAATGAGA-3’ |
| *Cd36* reverse | Sigma-Aldrich | 5’-CTTGTGTTTTGAACATTTCTGCTT-3’ |
| *Fgf21* forward | Sigma-Aldrich | 5’-CACCGCAGTCCAGAAAGTCT-3’ |
| *Fgf21* reverse | Sigma-Aldrich | 5’-GCAGGCCTCAGGATCAAAGT-3’ |
| *Adiponectin forward* | Sigma-Aldrich | 5’-GCAGAGATGGCACTCCTGGA-3’ |
| *Adiponectin reverse* | Sigma-Aldrich | 5’-GCAGAGATGGCACTCCTGGA-3’ |
| Resistin Forward | Sigma-Aldrich | 5’-GATAGACTGGACAGCAGCCC-3’ |
| Resistin Reverse | Sigma-Aldrich | 5’-GATAGACTGGACAGCAGCCC-3’ |
| HomArm *Cpt1a* loxP forward | Sigma-Aldrich | 5’-CAGGATCCCTTTGAGCAGCAG-3’ |
| HomArm *Cpt1a* loxP reverse: | Sigma-Aldrich | 5’-CAAAGTGGCCCCTAAGGCTAC-3’ |
| AgRP-CRE ER^T2^ forward: | Sigma-Aldrich | 5’-CAGATACCATCATCTCTCCC-3’ |
| AgRP-CRE ER^T2^ reverse | Sigma-Aldrich | 5’-CCTTAAACTCGCCCATATATGTGG-3’ |
| AgRP-CRE ER^T2^ control: | Sigma-Aldrich | 5’-GCTCTACTTCATCGCATTCCTTG-3’ |
| ZsGreen forward: | Sigma-Aldrich | 5’-AAAGTCGCTCTGAGTTGTTATCAG-3’ |
| ZsGreen reverse: | Sigma-Aldrich | 5’-GGAGCGGGAGAAATGGATATG-3’ |
| ZsGreen Control: | Sigma-Aldrich | 5’-TCACTGCATTCTAGTTGTGGTTTG-3’ |
| RiboTag loxP forward | Sigma-Aldrich | 5’-GGGAGGCTTGCTGGATATG-3’ |
| RiboTag loxP reverse: | Sigma-Aldrich | 5’-TTTCCAGACACAGGCTAAGTACAC-3’ |

Table S2

| **Antibodies** | **Dilution** | **Source** |  |
| --- | --- | --- | --- |
| Rabbit Anti-UCP-1 | 1:1000 | Abcam | Cat# ab10983 |
| Rabbit Anti-β-actin | 1:25,000 | Sigma-Aldrich | Cat# A3854 |
| Rabbit Anti-Fos | 1:200 | Cell signaling | Cat# 2250 |
| Rabbit Anti-GFP | 1:1000 | Abcam | Cat# ab290 |
| Rabbit Anti-HA | 1:1000 | BioLegend | Cat# MMS-101R |
| Goat Anti-Rabbit Alexa Fluor 488 | 1:1000 | Thermofisher | Cat# A-11008 |
| Goat Anti-Mouse IgG1 Alexa Fluor 568 | 1:1000 | Invitrogen | Cat#A21124 |
